# Network Engineering Using Autonomous Agents Increases Cooperation in Human Groups

**DOI:** 10.1016/j.isci.2020.101438

**Published:** 2020-08-06

**Authors:** Hirokazu Shirado, Nicholas A. Christakis

**Affiliations:** 1 School of Computer Science, Carnegie Mellon University, Pittsburgh, PA 15213, USA; 2 Yale Institute for Network Science, Yale University, New Haven, CT 06520, USA; 3 Department of Sociology, Yale University, New Haven, CT 06520, USA; 4 Department of Ecology & Evolutionary Biology, Yale University, New Haven, CT 06511, USA; 5 Department of Biomedical Engineering, Yale University, New Haven, CT 06520, USA

**Keywords:** Behavioral Neuroscience, Cognitive Neuroscience, Human-Computer Interaction, Collaborative Computing

## Abstract

Cooperation in human groups is challenging, and various mechanisms are required to sustain it, although it nevertheless usually decays over time. Here, we perform theoretically informed experiments involving networks of humans (1,024 subjects in 64 networks) playing a public-goods game to which we sometimes added autonomous agents (bots) programmed to use only local knowledge. We show that cooperation can not only be stabilized, but even promoted, when the bots intervene in the partner selections made by the humans, re-shaping social connections locally within a larger group. Cooperation rates increased from 60.4% at baseline to 79.4% at the end. This network-intervention strategy outperformed other strategies, such as adding bots playing tit-for-tat. We also confirm that even a single bot can foster cooperation in human groups by using a mixed strategy designed to support the development of cooperative clusters. Simple artificial intelligence can increase the cooperation of groups.

## Highlights

• Network experiments show that adding bots can promote cooperation in human groups
• To promote cooperation, bots intervene locally in the connections between humans
• Even a single bot, with simple AI, can foster cooperation by network engineering
• Network-engineering bots are effective even when identified as bots

## Graphical Abstract

**Figure:**
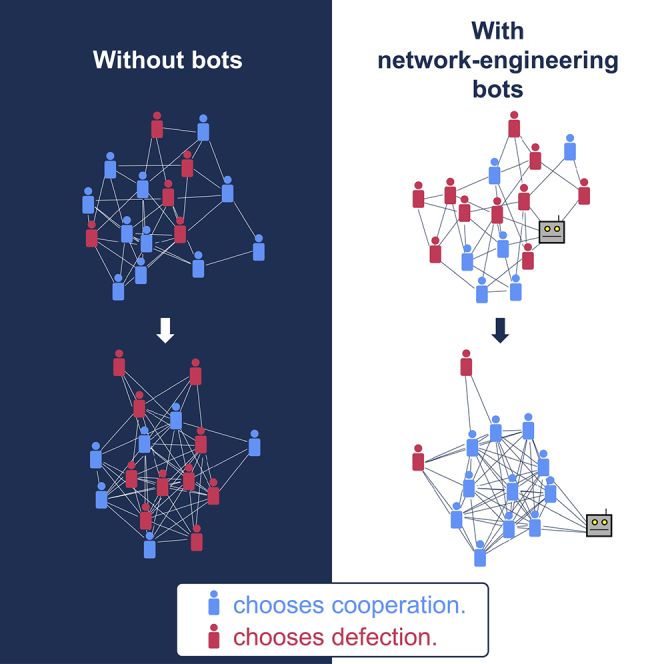

## Introduction

Human societies function best when people produce public goods that offer collective benefits that they could not otherwise obtain individually (Olson, 1965). The cooperation required to do this, however, is challenging because it creates a social dilemma (Axelrod, 1984; Dawes, 1980): the group does well if individuals cooperate, but, for each individual, there is a temptation to defect. Getting groups to cooperate, and to keep cooperating, therefore presents substantial difficulties (Hardin, 1968; Nowak, 2006).

Various strategies for individuals to minimize their own losses or to act fairly, from their own point of view, when facing a cooperation dilemma have been identified. And these actions might conceivably also sustain cooperation in groups as a whole. For instance, in a repeated game, individual strategies—such as Tit-For-Tat (Axelrod, 1984) and its variants (Hilbe et al., 2013; Nowak and Sigmund, 1993)—may lead another person to whom a person is connected to cooperate as a secondary effect. In fact, even robots can be programmed to elicit cooperation from humans (Crandall et al., 2018). Furthermore, a large body of work has explored broader, institutional approaches to overcoming cooperation dilemmas, such as reputation (Cuesta et al., 2015; Nowak and Sigmund, 2005), punishment (Fehr and Gachter, 2002; Fowler, 2005), rewards (Rand et al., 2009), population structure (Allen et al., 2017; Ohtsuki et al., 2006), tie rewiring (Rand et al., 2011), or the establishment of a central authority (Ostrom, 1990). But it is still unclear how a small fraction of individuals might guide a group of others toward the creation of public goods *without* a super-ordinate institutional change. And approaches that actually increase levels of cooperation in groups (from their baseline) are scant.

Here, we examine how individual autonomous agents acting locally can facilitate, and even increase, cooperation in a group of people. We focus on network interventions (Valente, 2012) on the assumption that all individuals, including those who attempt to intervene to make the situation better, are embedded in a social network and that their information and actions are limited to their local neighborhood of connections (Granovetter, 1985). We take preprogrammed autonomous agents endowed with various simple strategies (i.e., “bots” or computer programs behaving as individual actors in a social system) and introduce them into the network and have them interact as members of the group (Shirado and Christakis, 2017). We do not assume that the bots have distinctive advantages that allow them to observe and change the entire social system as a central authority might. As a result, we can (1) study the abstract principles instantiated by the bots (gaining insights into how similar actions initiated by humans might affect cooperation in groups, albeit with exquisite experimental control in the case of the bots) and (2) develop practical applications of distributed, simple artificial intelligence (AI) agents in the form of bots that might be introduced into online groups so as to modify their properties for the better (Paiva et al., 2018). In short, we explore how bots can be used to facilitate positive social outcomes.

Humans decide whether to cooperate in part based on the actions of their neighbors (Shirado et al., 2013). Thus, an intervening agent, which resembles what Axelrod has called a “reformer”(Axelrod, 1984) (here, one that it is embedded within the system as a participant itself), might, by exercising the behavioral options of cooperating or defection, not only affect its own payoffs but also have an impact on others. However, a reformer might be limited in its ability to make its own network environment favorable to cooperation (Liu et al., 2011). For instance, theory suggests that the simple strategy of continuous cooperation by a well-intentioned reformer may do little to change cooperation dynamics, especially in large groups (Olson, 1965), and any such cooperators might be solely exploited by the defectors connected to them (Axelrod, 1984).

To address the challenges faced by reformers embedded in social networks who seek to sustain or enhance group cooperation, we focus on network dynamics that allow individuals to adjust social ties and engage in a kind of social network engineering (Rand et al., 2011; Shirado et al., 2013). That is, we explore a strategy whereby each bot gives its human neighbor an option to make or break a tie with other human subjects chosen by the bot (the bots act as a kind of social assistant). To find an effective strategy for such reformer bots, we set up our experiments in two stages. In the first set of experiments, we examine various network interventions (including a variety of alternative, control strategies) using a sufficient number of bots (“Experiment 1”). We evaluate the efficacy of the intervention in a repeated set of interactions in a public goods game involving cooperation. In the second experiment, we specify the bot's intervention strategy based on the results of the first experiment and then test it with a single bot embedded in a group of human subjects (“Experiment 2”).

In Experiment 1, we recruited 896 unique human subjects through Amazon Mechanical Turk, dividing them among 56 groups, in sessions lasting an average of 24.1 min. Subjects were placed into groups of 16 individuals arranged in a network with an Erdős-Rényi random graph configuration (Erdős and Rényi, 1959) in which 30% of the possible ties were present at the outset, on average. In the sessions involving bots, each subject additionally received one connection with a bot. The subjects were therefore initially connected to an average 4.41 (SD = 1.76) other humans and 1 bot (i.e., 5.41 network neighbors on average). Subjects could identify each neighbor by a permanent, randomly generated name (see Data S1 and Transparent Methods).

Each subject played a public-goods game lasting 30 rounds with their network neighbors without knowing when the game would end. At the beginning of the game, subjects received US$1.00 as their initial endowment. In each round, all the subjects chose whether to cooperate, by reducing their own endowment US$0.05 per neighbor in order to increase the endowment of all their neighbors by US$0.10 each, or to defect, by paying no cost and providing no benefits. Subjects had to make the same choice with respect to all their connected neighbors (Allen et al., 2017; Cuesta et al., 2015; Ohtsuki et al., 2006; Rand et al., 2011, 2014; Shirado et al., 2013; Yamagishi, 1986).

After making their cooperation choice, subjects were informed of the choices made by their neighbors. Then, subjects had the opportunity to change their neighbors by making or breaking ties (“tie-rewiring” options), using a game setup that we had explored in prior experiments (Rand et al., 2011; Shirado et al., 2013). Specifically, 5% of all pairs of human subjects (i.e., 6 pairs, on average) were chosen at random in each round and were given the opportunity to rewire their ties. If a tie already existed between the two subjects, then one of the two was picked at random to be allowed to choose whether to voluntarily break the tie with the other; if a tie did not already exist between the two, a randomly selected member of the pair was given the option to form the tie (unilaterally). When making this decision, subjects were aware of whether the person to whom they might disconnect or connect had cooperated or defected in the past round. Thus, people could choose to modify a subset of their social ties (with low frequency) at each round; the network could become rewired as a result of these modifications; and all subjects' network properties (such as network degree and the fraction of cooperators among their neighbors) could change over time. Our focus was on the possible impact of network interventions with bots placed within these groups.

Within this basic setup, we introduced 16 bots into the network of 16 human subjects (except for the control sessions without any bots). Each bot had only one tie and was connected with a different subject at the beginning of the game (i.e., every subject initially had precisely one tie to a bot among their neighbors). Bots always chose cooperation in the game (except for the bots using the Tit-For-Tat strategy). And the bots never connected with each other.

To be clear, we used the artifice of single-tie bots for Experiment 1 so as to experimentally fix the total amount of intervention across sessions and treatments to 16 total ties with bots at all times and in all treatments. Sixteen single-tie bots corresponded to three human subjects in terms of average initial network degree, and the human-bot connections accounted for 11.8% of all the possible connections in a network (i.e., 16/136). Subjects were not informed that there were bots in the game (except for an extra condition making bots visible; see below).

Using the bots embedded in a network of human subjects, we tested the network-intervention strategy whereby each bot generated an additional rewiring option for its human neighbor (regardless of whether the neighbor cooperated or defected) to make or break a tie with other human subjects; the humans did not have to use the rewiring option given by the network-intervention bots. All the network-intervention strategies added the same amount of rewiring options to the social system (increasing the possible rewiring rate from 5% to 16%).

We then manipulated the criteria used by the bot reformers to pick targets. The bots intervened based on the cooperation decision that the humans had previously made. To improve the level of cooperation in a group using network engineering, an intervention would need not only to retain existing cooperators, but also to prompt defectors to change their decisions. Thus, we tested three processes, in terms of whether the approach is *random, engaged*, or *disengaged*—from the reformer's perspective—with respect to the *defectors* in the group (Rand et al., 2009). In the “random” process, targets are chosen at random. The target subject thus had an additional chance to form or break a tie with another subject, irrespective of the target subject's cooperation decision. In the “engaged” process, defectors are given a chance to form more cooperative ties so as to (hopefully) encourage them to switch to cooperation. The engaged process had every bot give an option to its neighbor to make a new tie with another subject who was choosing cooperation (so, for example, a defector connected to the bot was introduced to a cooperator). That is, the cooperative bots foster engagement with the defectors (i.e., the bots are conciliatory to defectors). In the “disengaged” process, the cooperative bots foster disengagement with defectors (i.e., the bots work to quarantine defectors from cooperators). The disengaged process involved the bot giving an option to its neighbor to cut an existing tie with another subject who was choosing defection.

We also conducted sessions with three types of control conditions. The first control condition did not involve any bots. In the other two control conditions, like the treatment conditions, the group of 16 human subjects had 16 single-tie bots in a network. In one control condition, the bots (again) always cooperated. In the other, they used the Tit-For-Tat strategy; they started with cooperation and then selected the same behavioral choice (i.e., cooperation or defection) as their human neighbor had selected in the previous round (Axelrod, 1984). Importantly, and in contrast to the treatments, the bots in these control conditions did *not* intervene in the local networks of the human subjects by giving them a rewiring option.

As noted, subjects were not informed that they were interacting with bots and that some of the tie-rewiring options were given by bots. To assess the effect of this ignorance, we also carried out experiments with an extra condition of “bot visible.” In this extra condition, subjects were informed that they were interacting with bots, which neighbors were bots, and which tie rewiring options the bots suggested them (see Data S1 and Transparent Methods). Thus, subjects could make a decision with knowledge that they had both bots and humans among their neighbors and that the bots gave them rewiring options. We examined this treatment only in the case in which the bots used the “disengaged” strategy.

In Experiment 2, which involved an additional 128 subjects in 8 sessions, we tested the minimal intervention of a single bot. This experimental setting differed from Experiment 1 only in that a group of 16 human subjects played a public-goods game with 1 bot having 5 ties, which corresponded to 1 average human subject in terms of initial network degree (in the sessions of Experiment 2, the average network degree = 5.13 at round 1). Thus, in contrast of Experiment 1, some subjects had a connection with the bot and the others did not (in every round). This single bot used a network-engineering strategy that we designed according to the results of Experiment 1 (as described below).

In sum, we evaluated the effect of bot interventions and network engineering with two experiments. Experiment 1 had 3 control conditions not involving any network-intervention bots, 3 treatment conditions (i.e., random, engaged, and disengaged criteria for how the bots treated defectors), and 1 extra condition involving manipulation of bot visibility. Experiment 2 had one condition involving a single bot. We conducted 8 sessions for each condition for a total of 64 groups with 1,024 human subjects in total.

Subjects interacted anonymously over the Internet using our publicly available software platform (available at breadboard.yale.edu). We allowed the participation only of those subjects who completed a tutorial session and passed a series of tests assessing their understanding of the game rules. We prohibited subjects from participating in more than one session. All subjects consented, and this study was approved by the Yale University Committee of the Use of Human Subjects.

## Results

### Improving Human Cooperation with Disengaged Intervention Bots (Experiment 1)

**Figure 1:**
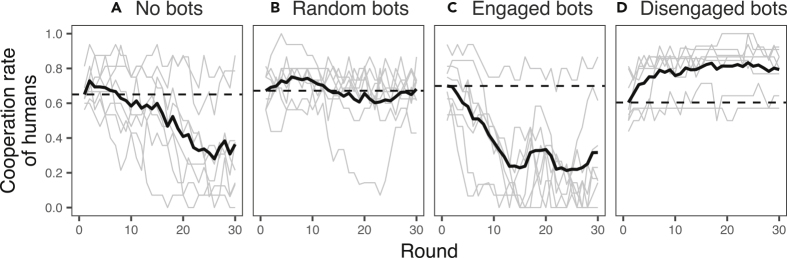
The Fraction of Cooperative Human Players per Round Light gray lines show results for each session, black lines show average across all experimental sessions for each treatment (*N*_*session*_ = 8 per treatment). Initial rates of average cooperation varied by chance across treatments (the dashed lines). See the results of two other control conditions (“always cooperate” and “tit-for-tat”) in Figure S1. Across all 48 groups, the average initial rate of cooperation was 68.2% ± 12.8%.

For the control sessions involving only human subjects (*N*_*subject*_ = 16 per session), on average, 65.1% of subjects per session started with choosing cooperation, and the fraction of cooperative players *decreased* over the rounds, eventually reaching 36.4% at the final round (p = 0.024; paired t test with *N*_*session*_ = 8 sessions; Figure 1A). This finding is in keeping with much prior work showing that defection overwhelms cooperation in human groups as interactions progress over time (Axelrod, 1984; Dawes, 1980; Nowak, 2006). These social dynamics favoring defection did not change materially when either the always-cooperative or Tit-For-Tat (TFT) bots were added to the group (Figure S1A). These findings illustrate the limited ability of such reformers' actions to help groups to maintain cooperation, whatever the relative value of these strategies for the actors themselves who play them. That is, TFT bots, for instance, did not help the groups in which they were embedded.

When bots using the network interventions were introduced to the group, the declining slope in cooperation depended on the criterion for rewiring that the bots used. When bots randomly gave tie-rewiring options to humans in their group (random intervention), the cooperation level of human subjects stayed steady (Figure 1B; 67.2% at the outset and 68.1% at the end, on average; p = 0.872; paired t test with *N*_*session*_ = 8). When bots gave the option for the humans to make a tie only to other subjects who chose cooperation (engaged intervention), cooperation decayed quickly from 69.9% and stabilized at 31.7% (Figure 1C; p < 0.001; paired t test with *N*_*session*_ = 8). In contrast, when bots gave the option to the humans to *break* a tie to other subjects choosing defection (disengaged intervention), cooperation *improved* from the initial status and stabilized at a high level of cooperation (Figure 1D; 60.4% at the outset and 79.4% at the end, on average; p = 0.020; paired t test with *N*_*session*_ = 8). We are not familiar with any prior work documenting interventions that have been able not just to achieve the maintenance of cooperation in groups involved in public goods scenarios, but also to effectuate an *increase* from the baseline rate of cooperation within a group, let alone using an approach involving network engineering with autonomous agents.

**Figure 2:**
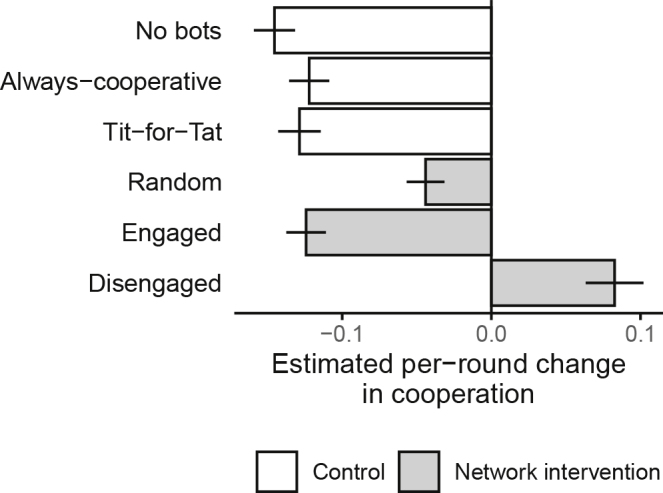
Average Change in Rates of Cooperation by Round Estimates based on GLMM, using a logistic regression model of individual cooperation choice with random effects for session and individuals (see Transparent Methods). The error bars are 95% confidence interval (CI).

We comprehensively evaluated how cooperation improved or decayed over the rounds across our six conditions. We conducted this analysis at the level of individual cooperation decisions using a generalized linear mixed model (GLMM) with random effects for sessions with nested individuals (see Transparent Methods). Figure 2 shows the relationship between the bot intervention strategy and individual-level cooperation. In regression models, only the network-intervention treatment using the disengaged (defector-detachment) criterion had a positive effect on the cooperation probability of individuals (p < 0.001; GLMM with *N*_*subject*_ = 768 in *N*_*session*_ = 48; 6 conditions × 8 sessions × 16 subjects). The other intervention strategies of the bots, such as TFT, failed to change the course of decision-making that otherwise favored defection in individuals across time (Figure 2). Moreover, a comparison of the disengaged strategy with each of the other strategies shows a significant improvement over those alternatives (p < 0.001; GLMM with *N*_*subject*_ = 768 in *N*_*session*_ = 48; see Table S1).

The overall average number of ties per human generally rose from the start in all conditions except for the sessions with disengaged intervention bots (Figure S1B). The disengaged intervention bots initially reduced the connections by offering tie-rewiring options to break connections from non-cooperative neighbors. Subjects did increase their connections and develop cooperative behavior after a couple of rounds, and the density reached the same level as that of the sessions without bots by the last round (p = 0.350; t test with *N*_*session*_ = 16); this also had implications for total contributions (Figure S1C).

**Figure 3:**
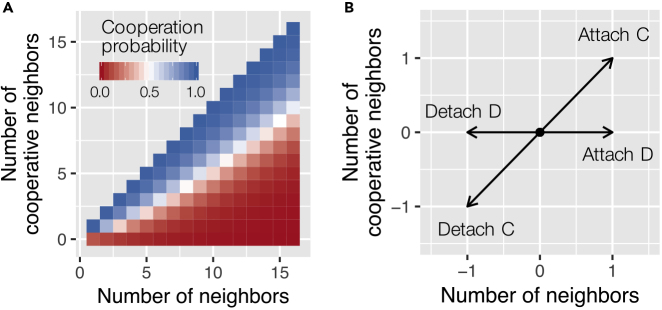
Cooperation Pattern with Neighborhood Change (A) Cell color shows cooperation probabilities estimated by GLMM shown in Table S2. (B) The impact of possible changes in a subject's surroundings is shown schematically. From a particular point in the parameter space (as shown in A), a person could move sideways or along the diagonal in the probabilistic space of human cooperation. Detaching or attaching to a defector changes the number of neighbors but does not change the number of cooperative neighbors; this direction obliquely crosses the contour of the cooperation probability distribution. On the other hand, detaching or attaching to a cooperator changes both the number of neighbors and the number of cooperative neighbors; such a change runs almost parallel to the contour of the cooperation probability distribution.

The disengaged intervention bots that fostered detachment from defectors helped subjects modify their neighborhood environment in ways that made it easier for the subjects to choose cooperation. We analyzed the network effects on cooperation decisions in human subjects using GLMM with random effects for individuals (see Transparent Methods for details). Figure 3A shows the estimated cooperation probability of a participant depending on the number of total neighbors and the number of cooperative neighbors (the interaction term also captures the *fraction* of cooperative neighbors). The individual-level analysis shows that network degree (i.e., the number of local neighbors) negatively affects the probability of a subject choosing cooperation, whereas the number and rate of cooperative neighbors have a positive impact on the cooperation probability (Table S2). In the setting of network interventions, adding or removing ties with defectors can cause a significant shift in individuals' cooperation probability (Figure 3B). Thus, the *engaged* intervention bots can discourage cooperators by helping defectors to attach to them (Figure 1C). On the other hand, the *disengaged* bots can encourage subjects (including even those who have chosen defection) to choose cooperation by helping them to detach from defectors (Figure 1D).

Furthermore, in our experimental conditions, only the disengaged intervention bots helped cooperators to *cluster* (Figure S1D). As the homophilic clusters of cooperators developed in the disengaged intervention condition, cooperators gradually outperformed defectors in terms of earnings (Figure S2). In the other treatments, the network dynamics were not enough to make cooperators outperform defectors (in terms of individual economic performance).

Using a further experiment, we confirmed that the effect of the disengaged intervention is obtained even when subjects knew whether they were interacting with bots, even when subjects knew which particular neighbors were bots, and even when subjects knew which tie rewiring options the bots had suggested to them (Figure S3). Subjects' awareness of bots and their interventions did not undermine the efficacy of the bots in this experimental setting.

### Substantial Impact of A Single Bot (Experiment 2)

The results of Experiment 1 suggest that successful interventions involving disengaged intervention bots may support the development of cooperative clusters. Thus, we tested a network-intervention strategy that was designed to support subjects to build cooperative clusters. In Experiment 2, we introduced 1 bot having 5 ties to a network of 16 human subjects (where the average degree was 5.13 at round 1).

**Figure 4:**
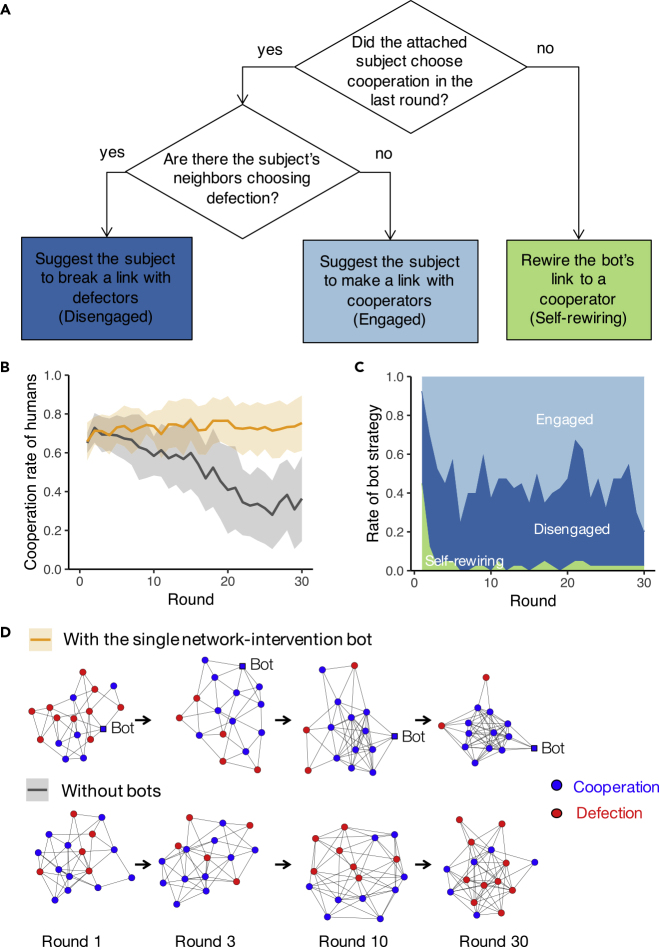
Cooperation and Network Dynamics with a Single Bot Deploying a Mixed Strategy (A) Control diagram for how a single bot intervenes in a network of human subjects. (B) Experiment results regarding average cooperation fraction with 95% CI (*N*_*session*_ = 8 for each treatment). The orange line indicates the result of sessions with the single network-engineering bot. The dark gray line indicates the result of sessions without bots, which is identical to Figure 1A. (C) Experiment results regarding average rate of the bot's intervention strategy actually applied to human players, over the rounds. (D) Network snapshots of an example session having a single bot and a session without bots.

This single bot deployed a slightly more complicated, mixed strategy (Figure 4A). If the bot's subjects (i.e., the humans to which it was directly connected) had chosen defection in the last round, the bot would detach and connect to other subjects who were cooperators in the last round (chosen at random). But if the bot's subjects had chosen cooperation in the last round, what the bot did next would depend on what the subjects' *neighbors* had previously done. If the subjects had a neighbor who had chosen defection, the bot suggested that the subject break their link to such a neighbor (or offered them the chance to do so), i.e., the disengaged strategy. (If the subjects had more than one neighbor who had chosen defection, the bot chose one of those neighbors at random to suggest that they be abandoned.) If, on the other hand, all of the subjects' neighbors were cooperators, then the bot offered the subjects the chance to connect to another cooperator (beyond the current neighbors the subjects had), i.e., the engaged strategy. (And this had the further result of increasing the number of connections the subjects had.)

As a result, this single bot substantially changed the social dynamics in these groups and improved cooperation in a network of 16 humans (Figure 4B; p < 0.001 with GLMM; *N*_*subject*_ = 128 in *N*_*session*_ = 8) while also fostering cooperative clusters (Figure S4). The bot initially sought out human partners through “self-rewiring” and then suggested to the humans to which it had connected that they rewire their ties (Figure 4C). Remarkably, the single bot eventually employed the engaged strategy more than 50% of the time in its working to improve cooperation, but the same engaged strategy considerably *diminished* cooperation when it was the sole strategy used from the outset of the interactions in Experiment 1 (Figure 1C). This indicates that it is the bot's adaptation of its intervention strategy to fit the humans' situation that is indeed efficiently driving the increase in cooperation. In parallel with the shift in strategies, the bot itself moved to the periphery of the network in the later rounds (Figure 4D). Given the initial interventions by the single bot, the humans came to foster cooperative circumstances *by themselves,* over time.

## Discussion

We find that embedded autonomous agents that are equipped with a simple kind of AI and that constitute a relatively small fraction of the connections in a group can act as network engineers and enhance collective cooperation. They achieve this by distancing individuals from non-cooperative members of their groups and by fostering clusters of cooperators within groups, both of which encourage people to become more cooperative. Although prior work has empirically shown the possibility of *maintaining* cooperation using diverse interventions, including that all human players be allowed to fully control their partner selections (Hauge et al., 2019; Rand et al., 2011; Shirado et al., 2013), our experiment shows a way of *promoting* cooperation, indeed with a decentralized, individually implemented strategy involving network engineering by autonomous agents. The cooperation level in the group actually rises from the initial condition when bots that undertake the controlled defector-excluding network intervention strategy are introduced (Figure 2). That is, with the disengaged bots, individuals who may have initially been on course to exploit others changed their mind, which rarely occurs in human-only groups (Peysakhovich et al., 2014; Shirado et al., 2013). Moreover, such tie brokerage in individuals strongly affects cooperation in the group even when people can see that it is bots, not humans, who are intervening in their connections (Figure S3).

Cutting ties to defectors in our experiments can be seen as a form of decentralized ostracism, here abetted by the bot network engineers. This approach requires no changes in the underlying interaction system or the imposition of a central authority with a view of the whole system. The distributed bots neither consolidate information nor enforce rules. It builds on experiments with dynamic networks, where participants make their own decisions about whom to exclude from the benefits of individual cooperation (Rand et al., 2011; Shirado et al., 2013). We find that network-wide cooperation can indeed be increased using autonomous agents, including even a single individual agent that evolves its intervention strategies and leverages human behaviors regarding partner selection (Figure 4).

Models of evolutionary dynamics suggest that evolution may favor cooperative types when partner choice is feasible (Eshel and Cavalli-Sforza, 1982; McNamara et al., 2008; Stanley et al., 1993), and evidence from Hadza foragers (and other groups) suggests that people are strongly influenced in their cooperation behavior by the norms of the groups to which they belong (Smith et al., 2018). By endowing actors with the tendency to manipulate the ties around themselves, bots (and people) may be able to affect the cooperativity of their groups, creating a convivial local environment for cooperative behavior. It is known that the ties between an ego's neighbors (i.e., network transitivity) play an essential role in collective action (Centola and Macy, 2007). And the clustering of cooperators, whether induced by intervention (Rand et al., 2014) or occurring naturally (Apicella et al., 2012), is known to facilitate cooperation. Hence, personal introductions might be an important mechanism in the evolutionary process favoring cooperation (Nowak, 2006) and might play a role in confronting collective action problems (Olson, 1965). It is possible that humans might have required third-party mediation in their tie formation in parallel with evolving the capacity for cooperation (Purzycki et al., 2016), and there is some evidence that interpersonal variation in network transitivity may be heritable (Fowler et al., 2009). By engineering the tie formation between humans, autonomous agents can therefore help humans develop favorable environments for cooperation.

Our work indicates that it is possible to use simple computer programs to have meaningful effects on collective behavior because, in the social situations that concern us, the bots are mixed in with (much smarter) humans on a level playing field in what we call “hybrid systems” (Paiva et al., 2018; Rahwan et al., 2019; Shirado and Christakis, 2017; Traeger et al., 2020). The bots can function as a kind of social catalyst, helping humans to help themselves. Artificial-intelligence agents that are being developed to enhance the social good might not need to be extremely sophisticated, nor might they need to replace human cognition. Rather, they need only supplement human interaction. Since humans can change their own code of behavior by perceiving other people's behaviors (Axelrod, 1984), the simplicity and transparency of decision-making in artificial agents might make it even more intelligible to humans, thereby eliciting effective and stable effects (Nass et al., 1994).

Our work sheds light on techniques that might maintain or even increase rates of cooperation in groups, and it also offers the prospect of practical applications in online networks, where bot technology based on people recommendation might be useful (Guy, 2018). For instance, it might be possible to improve the behavior of groups engaged in collective tasks like editing online materials (Kittur et al., 2007) or to reduce online harassment in social media (Johnson et al., 2019; Munger, 2017). On the other hand, bot interventions that result in high levels of transitivity might increase group-think and echo chamber effects (Stewart et al., 2019).

Our study offers empirical evidence that adding a few connections with autonomous agents in the role of reformers, mixed into human groups, can promote cooperation via a kind of network engineering. Although the results of laboratory experiments do not translate directly into the real world, the evidence suggests that agents that strategically intervene in local tie formation might actually increase cooperation in social networks—a notoriously difficult thing to do. Simple forms of artificial intelligence offer the prospect of improving human social interactions within groups.

### Limitations of the Study

Our work involves subjects interacting online in a highly stylized way. Moreover, there are features potentially relevant to cooperation interventions that our experiments do not explore, for example: how individuals would behave if they could choose cooperation separately for each partner (Melamed et al., 2018); whether individuals might recognize partners' attributes other than their cooperativity (Nishi et al., 2015); how accurate the information the bots rely on must be (Waniek et al., 2018); or whether the payoff structure (Ohtsuki et al., 2006), group size (Nosenzo et al., 2013), network topology (Allen et al., 2017), or the fraction and precise position of bot connections matter (Liu et al., 2011; Masuda, 2012). Not only agents' strategy but also agents' appearance and expression could affect human cooperation decisions (de Melo et al., 2018; Ju and Leifer, 2008). Another promising topic is developing more sophisticated approaches for the bots to learn the interaction strategies of humans (e.g., using deep learning techniques) (Vinyals et al., 2019). This study suggests that bots could influence social dynamics in human groups not only through their behavioral responses (typified by Tit-for-Tat and its variants) (Crandall et al., 2018), but also through their interventions in the social connections between people (Guy, 2018). Here, we only used simple algorithm in each bot. Bots might improve human cooperation more efficiently when they take a more flexible approach to adapt their intervention strategy to both individual and group situations. These are all important directions for future work.

### Resource Availability

#### Lead Contact

Further information and requests for resources should be directed to and will be fulfilled by the Lead Contact, Hirokazu Shirado (shirado@cmu.edu).

#### Material Availability

This study did not generate new unique materials.

#### Data and Code Availability

The data and code in this manuscript are available at Mendeley Data: [https://doi.org/10.17632/t963ktp6ft.1].

## Methods

All methods can be found in the accompanying Transparent Methods supplemental file.

## Supplemental Information

Document S1. Transparent Methods, Figures S1–S4, Tables S1 and S2, and Data S1

## References

[bib1] Allen B., Lippner G., Chen Y.-T., Fotouhi B., Momeni N., Yau S.-T., Nowak M.A. (2017). Evolutionary dynamics on any population structure. Nature.

[bib2] Apicella C.L., Marlowe F.W., Fowler J.H., Christakis N.A. (2012). Social networks and cooperation in hunter-gatherers. Nature.

[bib3] Axelrod R. (1984). The Evolution of Cooperation.

[bib4] Centola D., Macy M. (2007). Complex contagions and the weakness of long ties. Am. J. Sociol..

[bib5] Crandall J.W., Oudah M., Tennom, Ishowo-Oloko F., Abdallah S., Bonnefon J.-F., Cebrian M., Shariff A., Goodrich M.A., Rahwan I. (2018). Cooperating with machines. Nat. Commun..

[bib6] Cuesta J.A., Gracia-Lázaro C., Ferrer A., Moreno Y., Sánchez A. (2015). Reputation drives cooperative behaviour and network formation in human groups. Sci. Rep..

[bib7] Dawes R.M. (1980). Social dilemmas. Annu. Rev. Psychol..

[bib8] de Melo, C.M., Khooshabeh, P., Amir, O., and Gratch, J. (2018). Shaping Cooperation between Humans and Agents with Emotion Expressions and Framing. In Proceedings of the 17th International Conference on Autonomous Agents and Multi Agent Systems, 2224–2226.

[bib9] Erdős P., Rényi A. (1959). On random graphs. Publ. Math..

[bib10] Eshel I., Cavalli-Sforza L.L. (1982). Assortment of encounters and evolution of cooperativeness. Proc. Natl. Acad. Sci. U.S.A.

[bib11] Fehr E., Gachter S. (2002). Altruistic punishment in humans. Nature.

[bib12] Fowler J.H. (2005). Altruistic punishment and the origin of cooperation. Proc. Natl. Acad. Sci. U.S.A.

[bib13] Fowler J.H., Dawes C.T., Christakis N.A. (2009). Model of genetic variation in human social networks. Proc. Natl. Acad. Sci. U.S.A.

[bib14] Granovetter M. (1985). Economic action and social structure: the problem of embeddedness. Am. J. Sociol..

[bib15] Guy I., Brusilovsky P., He D. (2018). People recommendation on social media.

[bib16] Hardin G. (1968). The tragedy of the commons. Science.

[bib17] Hauge K.E., Brekke K.A., Nyborg K., Lind J.T. (2019). Sustaining cooperation through self-sorting: the good, the bad, and the conditional. Proc. Natl. Acad. Sci. U.S.A.

[bib18] Hilbe C., Nowak M.A., Sigmund K. (2013). Evolution of extortion in iterated prisoner’s dilemma games. Proc. Natl. Acad. Sci. U.S.A.

[bib19] Johnson N.F., Leahy R., Restrepo N.J., Velasquez N., Zheng M., Manrique P., Devkota P., Wuchty S. (2019). Hidden resilience and adaptive dynamics of the global online hate ecology. Nature.

[bib20] Ju W., Leifer L. (2008). The design of implicit interactions: making interactive systems less obnoxious. Des. Issues.

[bib21] Kittur A., Suh B., Pendleton B.A., Chi E.H. (2007). He says, she says: conflict and coordination in Wikipedia. SIGCHI.

[bib22] Liu Y.-Y., Slotine J.-J., Barabási A.-Á. (2011). Controllability of complex networks. Nature.

[bib23] Masuda N. (2012). Evolution of cooperation driven by zealots. Sci. Rep..

[bib24] McNamara J.M., Barta Z., Fromhage L., Houston A.I. (2008). The coevolution of choosiness and cooperation. Nature.

[bib25] Melamed D., Harrell A., Simpson B. (2018). Cooperation, clustering, and assortative mixing in dynamic networks. Proc. Natl. Acad. Sci. U S A.

[bib26] Munger K. (2017). Tweetment effects on the tweeted: experimentally reducing racist harassment. Polit. Behav..

[bib27] Nass C., Steuer J., Tauber E.R. (1994). Computers are social actors. SIGCHI.

[bib28] Nishi A., Shirado H., Rand D.G., Christakis N.A. (2015). Inequality and visibility of wealth in experimental social networks. Nature.

[bib29] Nosenzo D., Quercia S., Sefton M. (2013). Cooperation in small groups: the effect of group size. Exp. Econ..

[bib30] Nowak M., Sigmund K. (1993). A strategy of win-stay, lose-shift that outperforms tit-for-tat in the Prisoner's Dilemma game. Nature.

[bib31] Nowak M.A. (2006). Evolutionary Dynamics.

[bib32] Nowak M.A., Sigmund K. (2005). Evolution of indirect reciprocity. Nature.

[bib33] Ohtsuki H., Hauert C., Lieberman E., Nowak M.A. (2006). A simple rule for the evolution of cooperation on graphs and social networks. Nature.

[bib34] Olson M. (1965). The Logic of Collective Action: Public Goods and the Theory of Groups.

[bib35] Ostrom E. (1990). Governing the Commons.

[bib36] Paiva A., Santos F.P., Santos F.C. (2018). Engineering pro-sociality with autonomous agents. AAAI.

[bib37] Peysakhovich A., Nowak M.A., Rand D.G. (2014). Humans display a “cooperative phenotype” that is domain general and temporally stable. Nat. Commun..

[bib38] Purzycki B.G., Apicella C., Atkinson Q.D., Cohen E., McNamara R.A., Willard A.K., Xygalatas D., Norenzayan A., Henrich J. (2016). Moralistic gods, supernatural punishment and the expansion of human sociality. Nature.

[bib39] Rahwan I., Cebrian M., Obradovich N., Bongard J., Bonnefon J.-F., Breazeal C., Crandall J.W., Christakis N.A., Couzin I.D., Jackson M.O. (2019). Machine behaviour. Nature.

[bib40] Rand D.G., Arbesman S., Christakis N.A. (2011). Dynamic social networks promote cooperation in experiments with humans. Proc. Natl. Acad. Sci. U S A.

[bib41] Rand D.G., Dreber A., Ellingsen T., Fudenberg D., Nowak M.A. (2009). Positive interactions promote public cooperation. Science.

[bib42] Rand D.G., Nowak M.A., Fowler J.H., Christakis N.A. (2014). Static network structure can stabilize human cooperation. Proc. Natl. Acad. Sci. U S A.

[bib43] Shirado H., Christakis N.A. (2017). Locally noisy autonomous agents improve global human coordination in network experiments. Nature.

[bib44] Shirado H., Fu F., Fowler J.H., Christakis N.A. (2013). Quality versus quantity of social ties in experimental cooperative networks. Nat. Commun..

[bib45] Smith K.M., Larroucau T., Mabulla I.A., Apicella C.L. (2018). Hunter-Gatherers maintain assortativity in cooperation despite high levels of residential change and mixing. Curr. Biol..

[bib46] Stanley E., Ashlock D., Tesfatsion L. (1993). Iterated Prisoner's Dilemma with Choice and Refusal of Partners. https://lib.dr.iastate.edu/econ_las_economicreports/9.

[bib47] Stewart A.J., Mosleh M., Diakonova M., Arechar A.A., Rand D.G., Plotkin J.B. (2019). Information gerrymandering and undemocratic decisions. Nature.

[bib48] Traeger M.L., Strohkorb Sebo S., Jung M., Scassellati B., Christakis N.A. (2020). Vulnerable robots positively shape human conversational dynamics in a human-robot team. Proc. Natl. Acad. Sci. U S A.

[bib49] Valente T.W. (2012). Network interventions. Science.

[bib50] Vinyals O., Babuschkin I., Czarnecki W.M., Mathieu M., Dudzik A., Chung J., Choi D.H., Powell R., Ewalds T., Georgiev P. (2019). Grandmaster level in StarCraft II using multi-agent reinforcement learning. Nature.

[bib51] Waniek M., Michalak T.P., Wooldridge M.J., Rahwan T. (2018). Hiding individuals and communities in a social network. Nat. Hum. Behav..

[bib52] Yamagishi T. (1986). The provision of a sanctioning system as a public good. J. Personal. Soc. Psychol..

