# Supplementary material for: Network Engineering Using Autonomous Agents Increases Cooperation in Human Groups
Source: iScience. 2020 Aug 6;23(9):101438. doi: 10.1016/j.isci.2020.101438 (PMC7452167; doi:10.1016/j.isci.2020.101438)
Supplement: Document S1. Transparent Methods, Figures S1–S4, Tables S1 and S2, and Data S1 [file mmc1.pdf]

iScience, Volume *23*

Supplemental Information

Network Engineering Using Autonomous Agents Increases Cooperation in Human Groups

Hirokazu Shirado and Nicholas A. Christakis

Supplementary Information

1. Supplementary materials
2. Transparent methods
3. Supplementary references

1. Supplementary materials

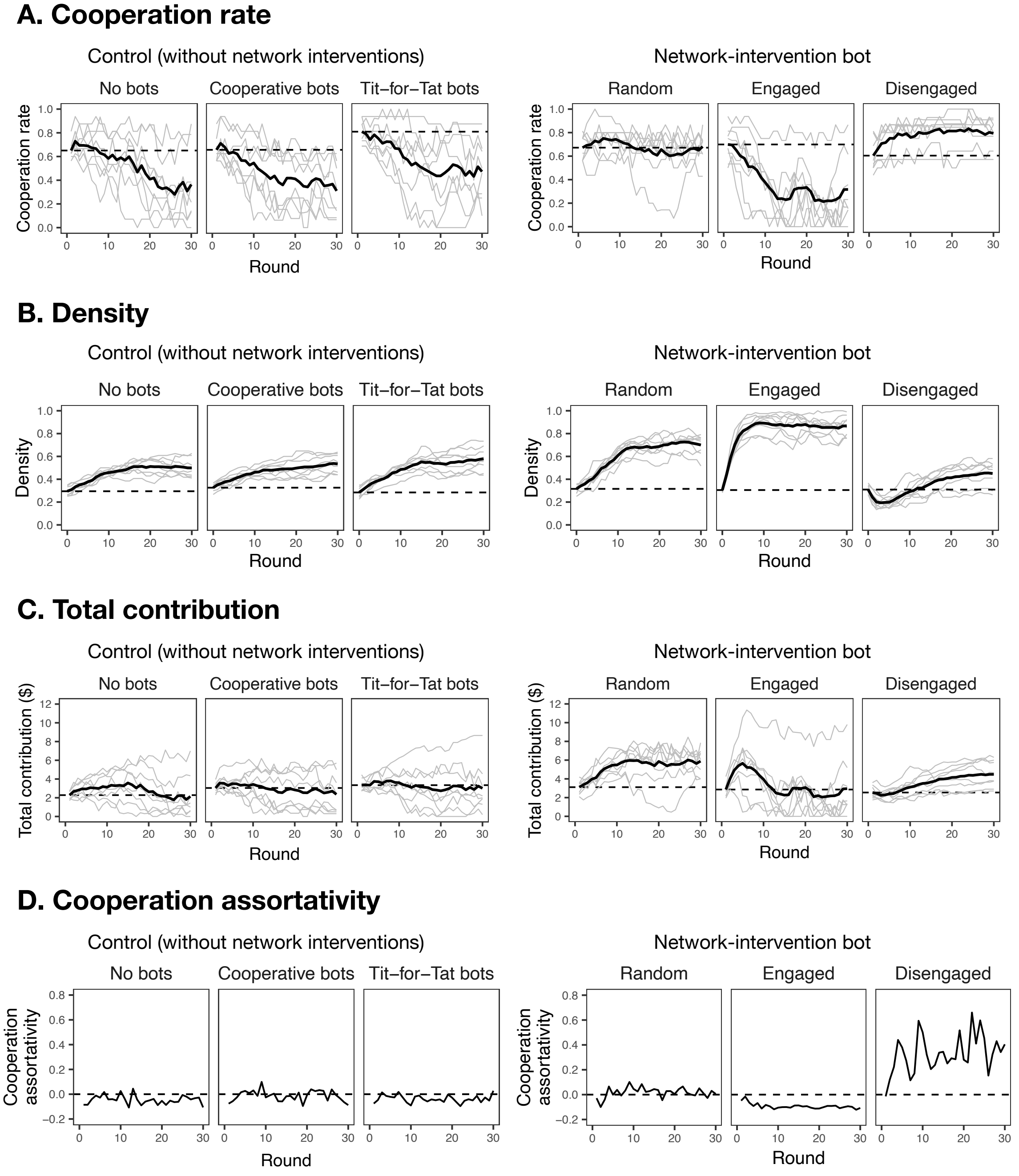

Figure S1. Experiment results, Related to Figure 1. (A) The fraction of cooperative human subjects by round. (B) Network density of human subjects by round. (C) Total amount of contribution from human subjects by round. Light gray lines show results for each session, black lines show average across all experimental sessions ($N_{session}$=8 per treatment). Dashed lines show the initial average value per treatment. (D) Assortativity for cooperation in a human group by round. Cooperation assortativity is quantified by the correlation between pairs of connected nodes in terms of cooperation choice. Dashed lines show 0 coefficient of assortativity that indicates the same level of random connections regarding the behavioral choice.

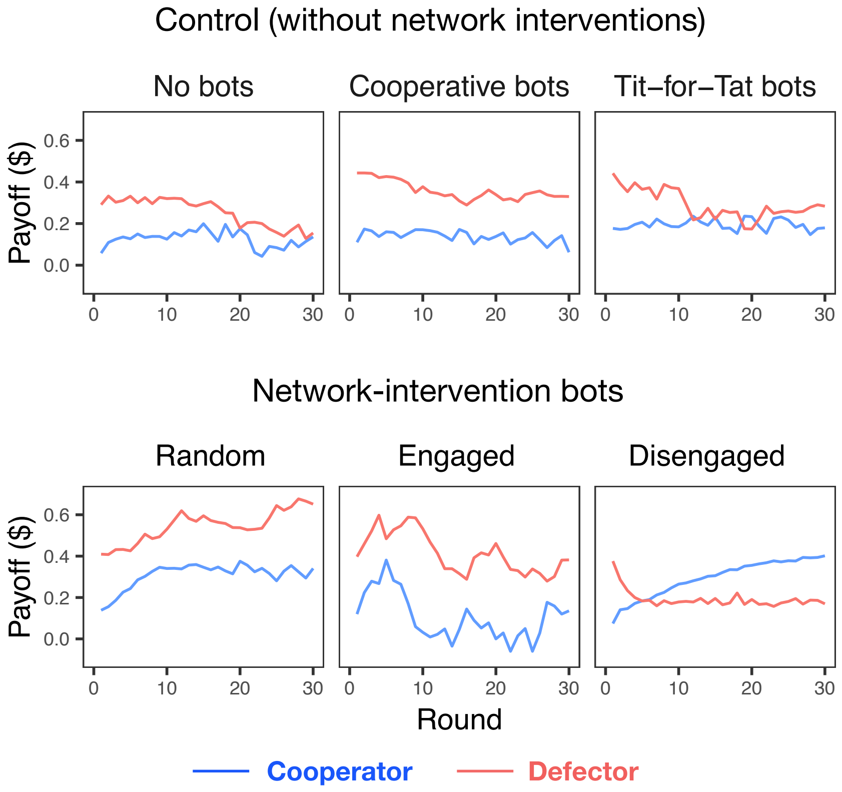

Figure S2. Per-round payoff of human players by cooperation choice, Related to Figure 1. Blue lines show the average payoffs that cooperating subjects received at the indicated round; red lines show those of defecting subjects.

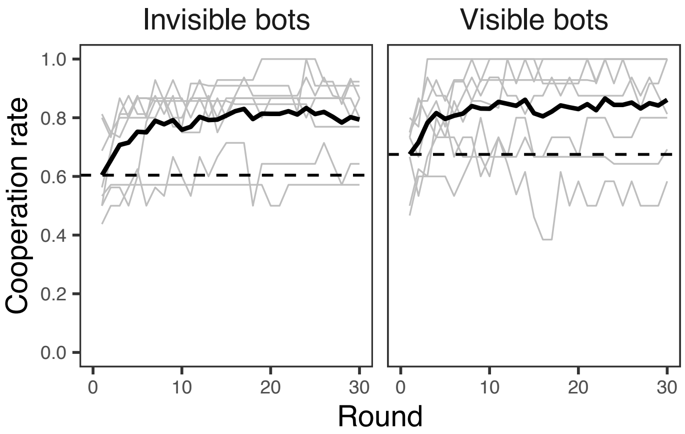

Figure S3. Fraction of cooperative human players by bot visibility, Related to Figure 1. Light gray lines show results for each session, black lines show average across all experimental sessions for each treatment ($N_{session}$=8 per treatment), and dashed lines show the initial rates of average cooperation. In the sessions with visible bots, humans were informed of which nodes were played by bots and which rewiring options were suggested by bots. Int the session with invisible bots, humans were not informed (which is identical to Figure 1D). Bots intervened with the disengaged network-intervention strategy in both conditions.

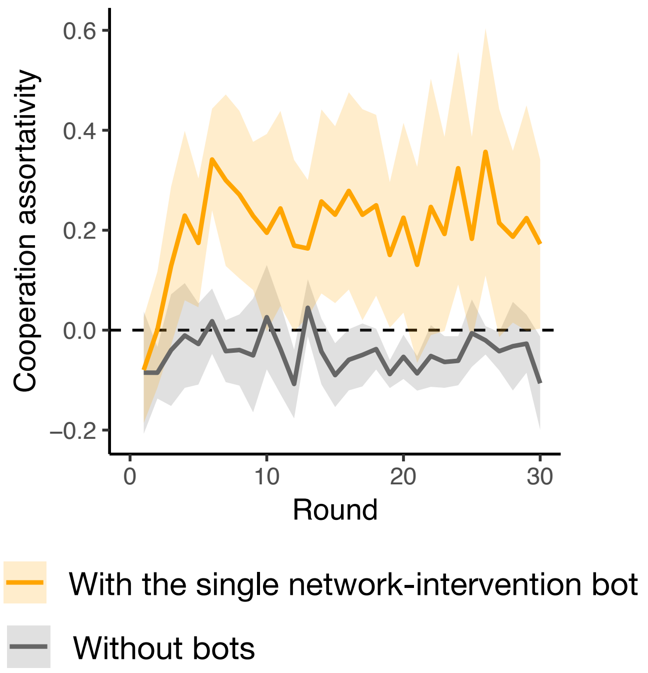

Figure S4. Change of cooperation assortativity in groups with a single bot using a mixed strategy, Related to Figure 4. The shades indicate 95% C.I. ($N_{session}$=8)

Table S1. The results of the statistical analysis regarding per-round cooperation change across bot treatments, estimated by GLMM with logit model incorporating random effects for sessions and individuals, Related to Figure 2.

Intercept…………………………………………………	2.122	***
	(0.300)	
Round…………………………………………………	0.083	***
	(0.010)	
Round × Bot treatment (ref. Disengaged bots)		
No bots…………………………………………………	-0.228	***
	(0.012)	
Always-cooperative bots……………………………	-0.205	***
	(0.012)	
Tit-for-Tat bots………………………………………	-0.211	***
	(0.012)	
Random bots…………………………………………	-0.127	***
	(0.12)	
Engaged bots…………………………………………	-0.207	***
	(0.12)	
Number of observations……………………………	21146	

NOTE. Clustered standard errors are given in parentheses.
*** $P < 0.01$; ** $P < 0.05$; * $P < 0.1$

Table S2. The results of the statistical analysis regarding cooperation probability, estimated by GLMM with logit model incorporating random effects for individuals, Related to Figure 3.

	Model without status-quo effect		Model with status-quo effect	
Intercept	1.221	***	0.797	***
	(0.187)		(0.175)	
Environement: Number of neighbors	-1.150	***	-1.073	***
	(0.216)		(0.202)	
Fraction of cooperators in neighbors	1.580	***	1.485	***
	(0.258)		(0.241)	
Number of cooperators in neighbors	1.216	***	1.065	***
	(0.327)		(0.307)	
Round	-0.393	***	-0.312	***
	(0.038)		(0.038)	
Self-action: From cooperation			0.593	***
(ref. from defection)			(0.068)	
Number of observations	20389		20389	

NOTE1. The environment covatiates and round number are standarized for estimation covergence.
NOTE2. Clustered standard errors are given in parentheses.
NOTE3. The data does not include the first round.
*** $P < 0.01$; ** $P < 0.05$; * $P < 0.1$

Data S1. Instruction and tutorials, Related to Figure 1. Below are screenshots for the initial description of the tutorial and the confirmation tests. We also show example screenshots of a real game.

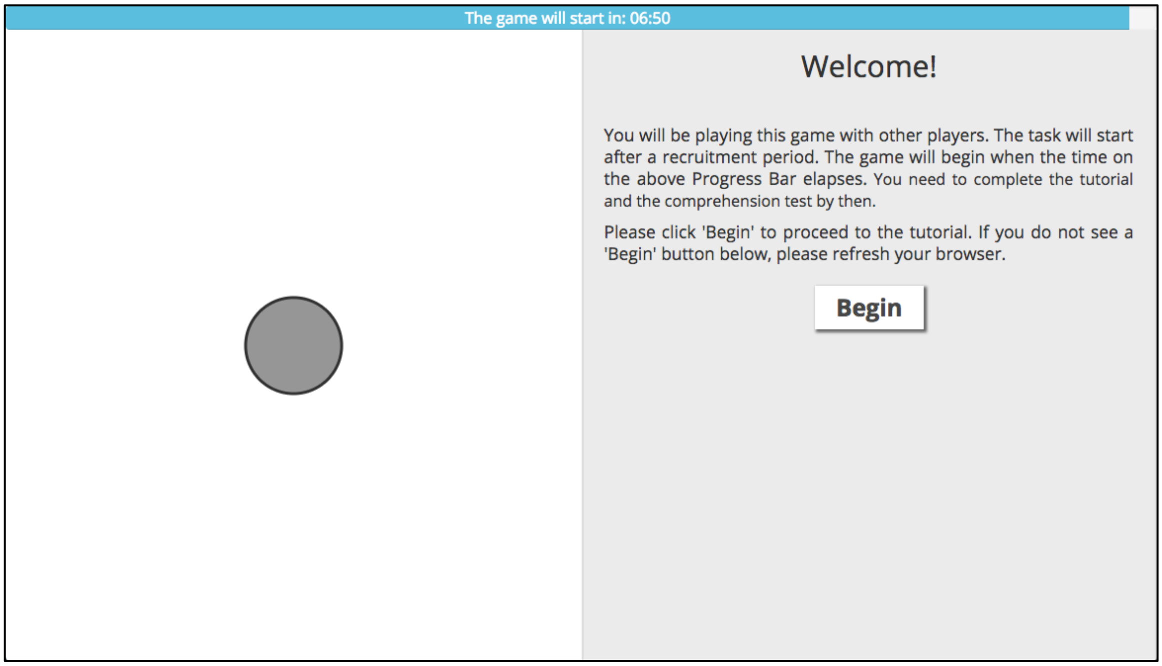

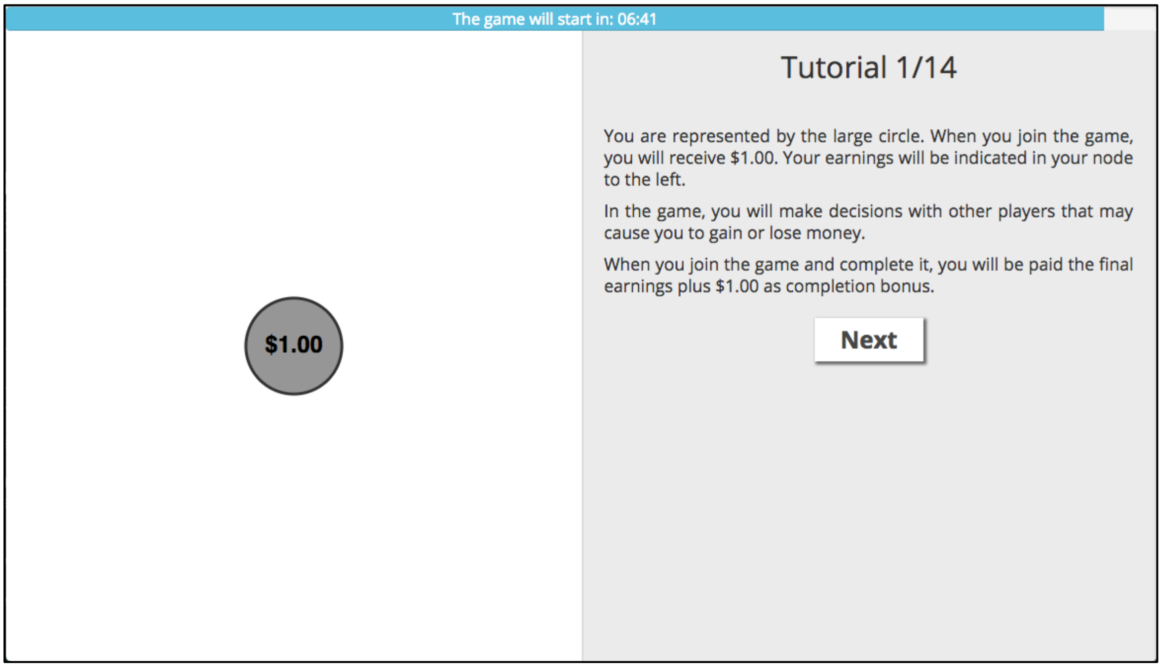

Data S1. Instruction and tutorials, Related to Figure 1. (cont.)

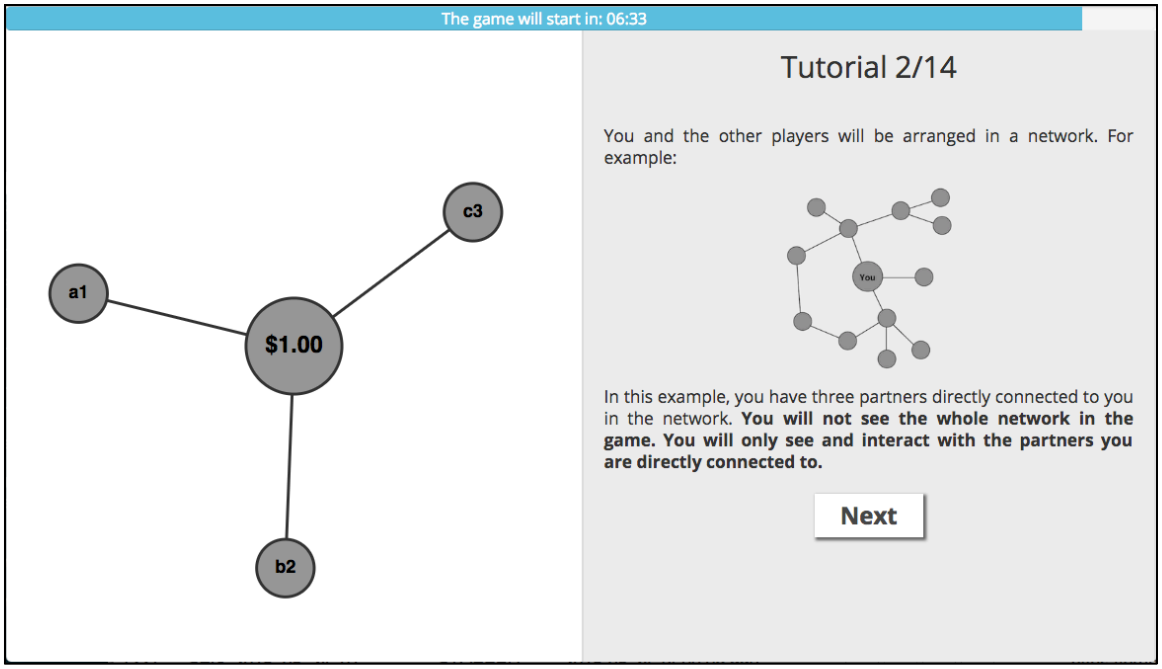

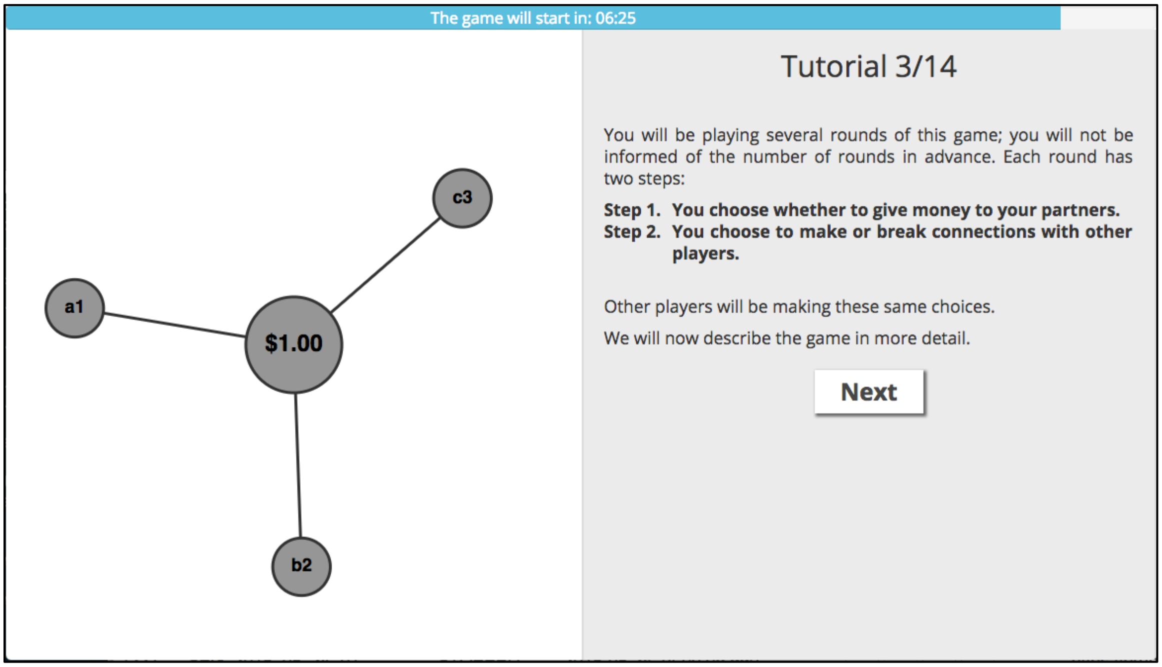

Data S1. Instruction and tutorials, Related to Figure 1. (cont.)

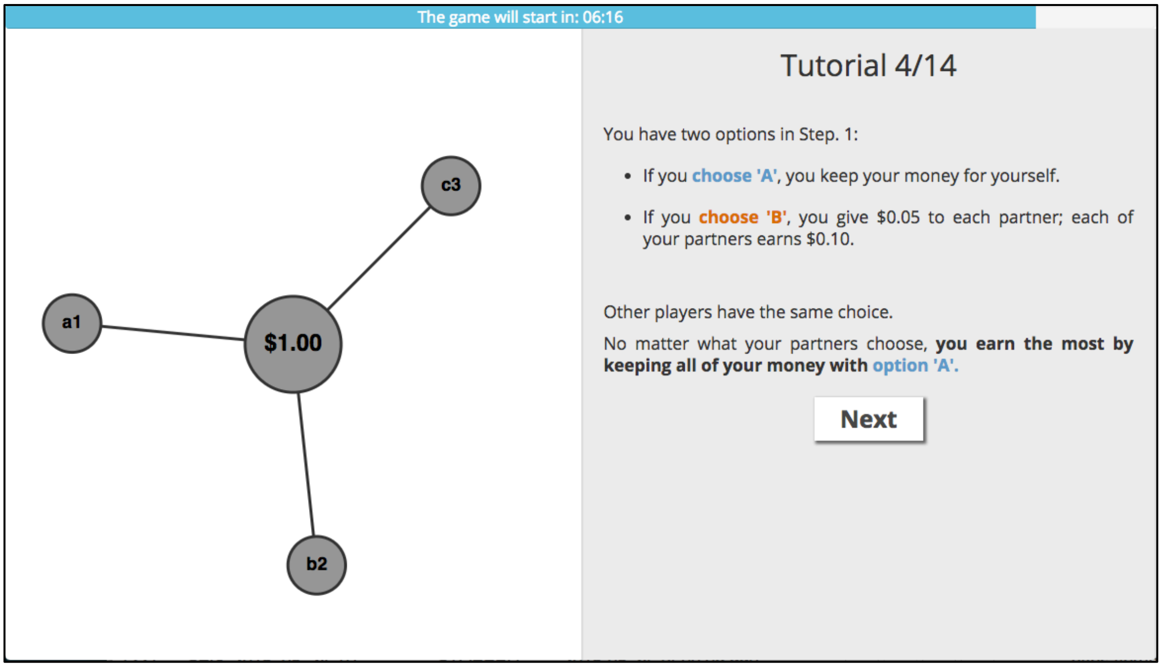

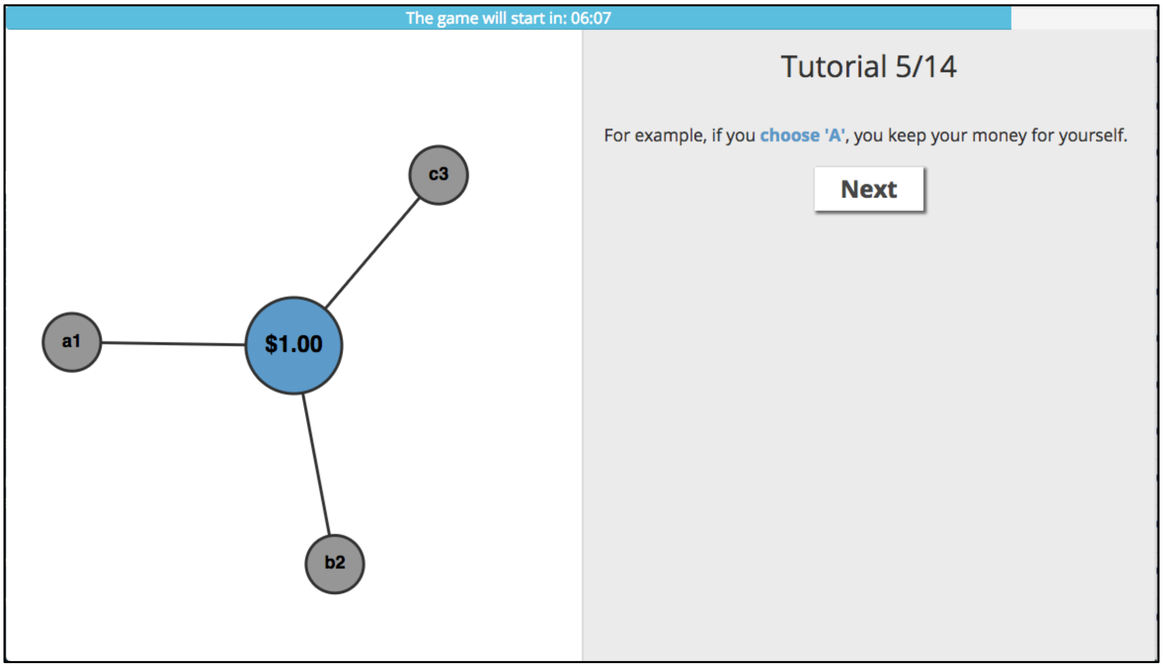

Data S1. Instruction and tutorials, Related to Figure 1. (cont.)

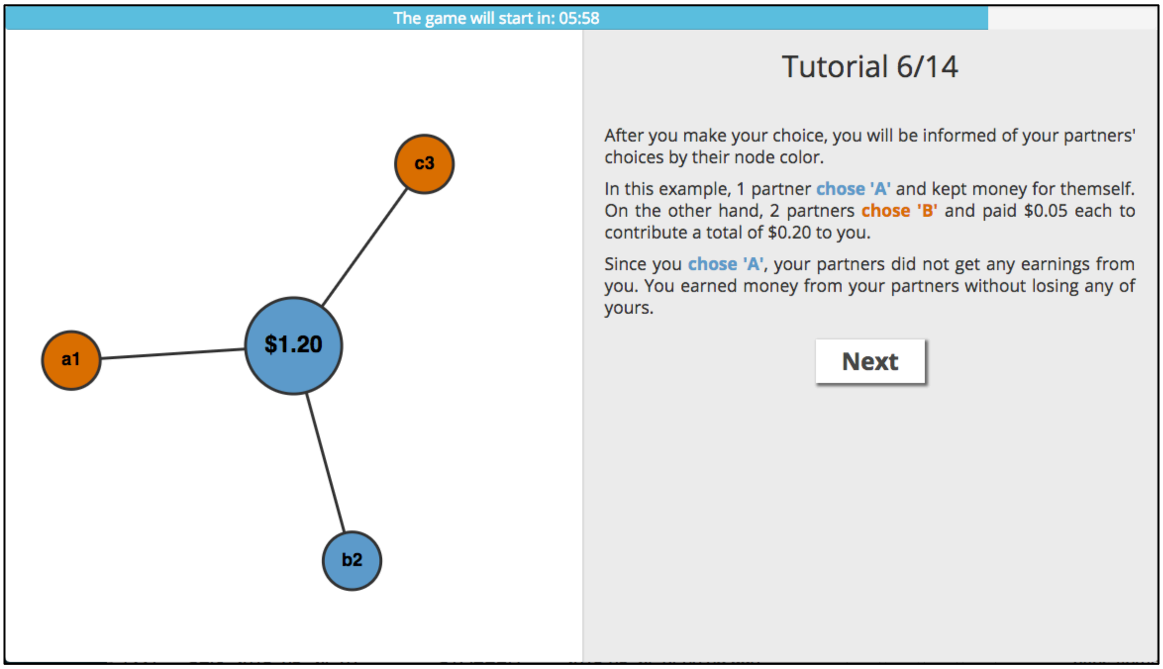

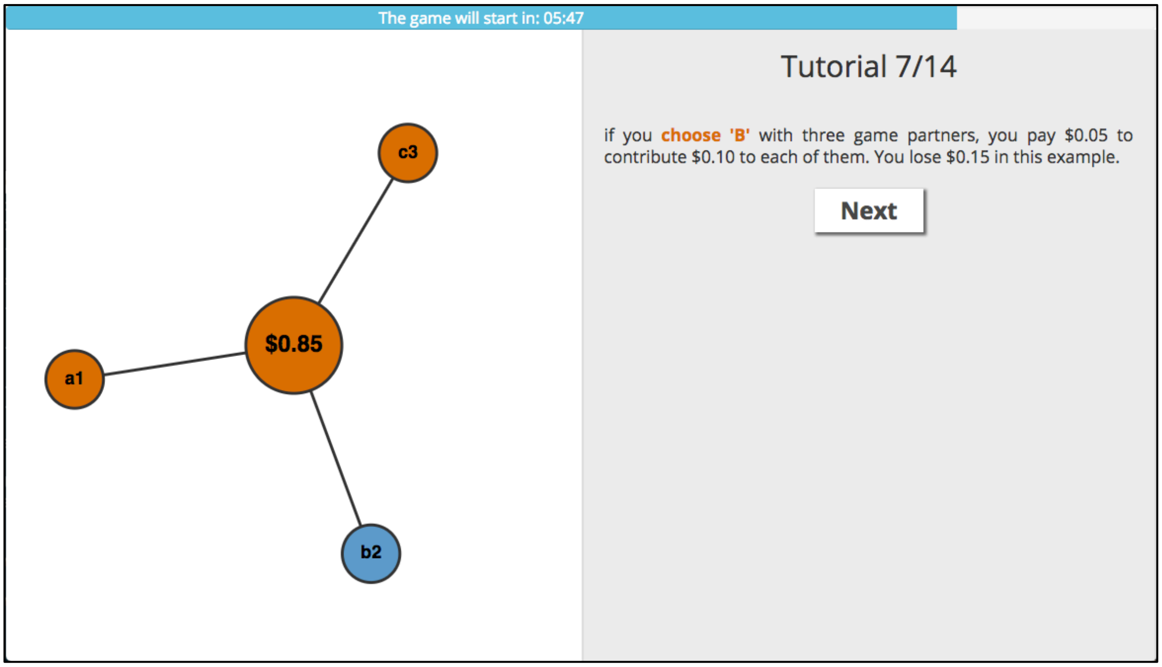

Data S1. Instruction and tutorials, Related to Figure 1. (cont.)

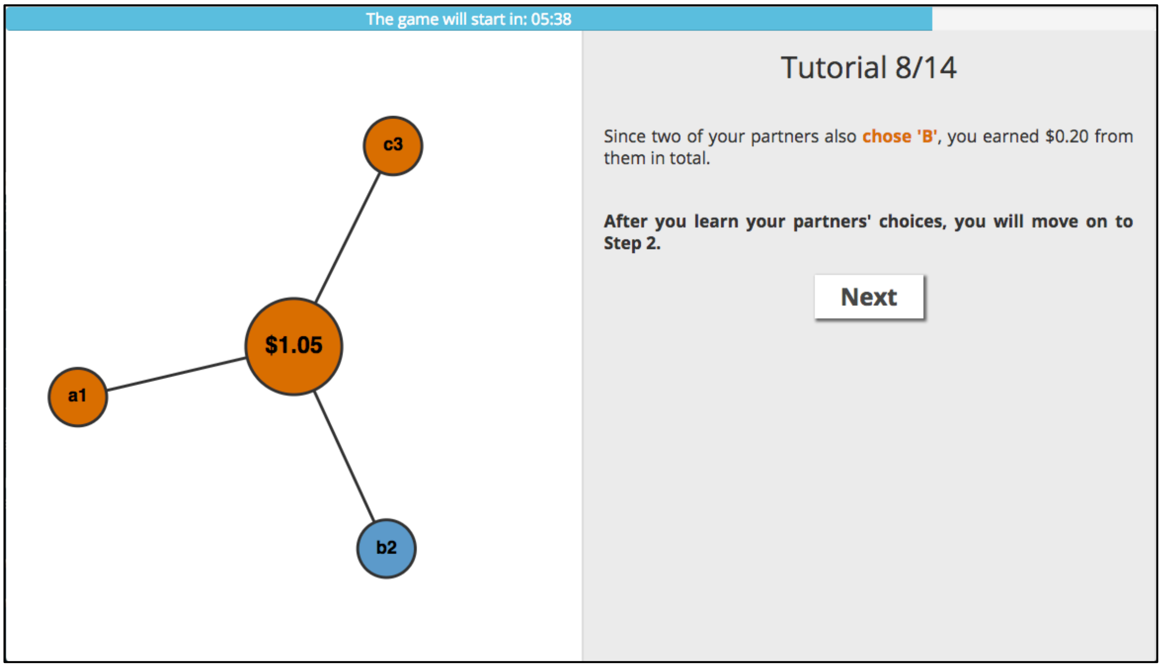

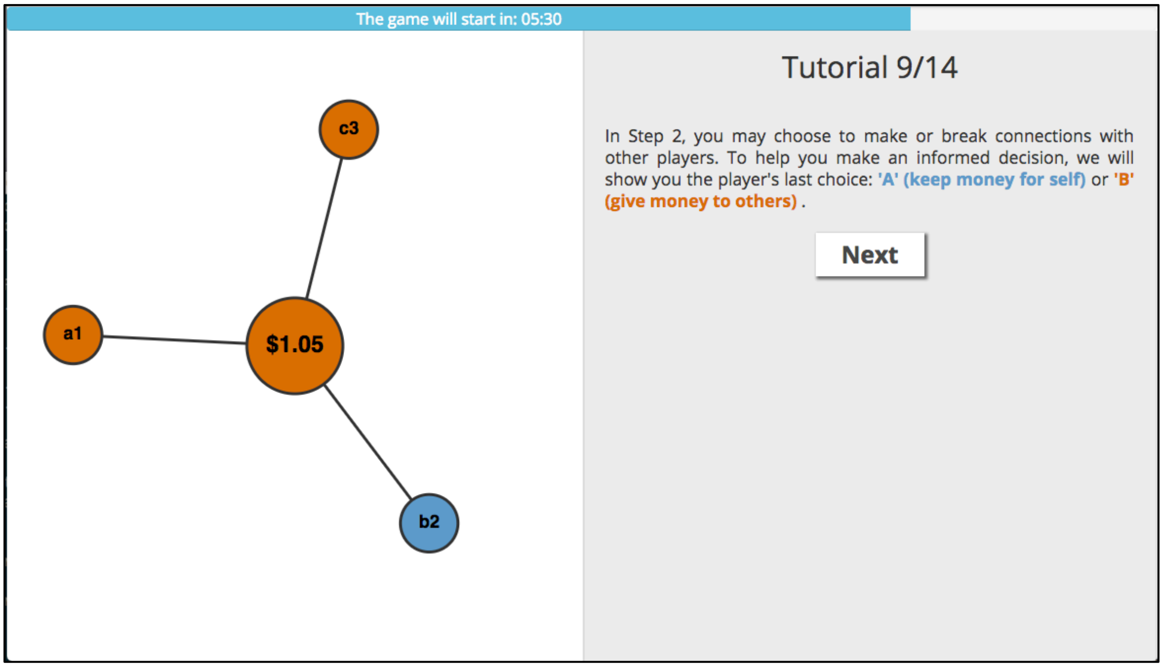

Data S1. Instruction and tutorials, Related to Figure 1. (cont.)

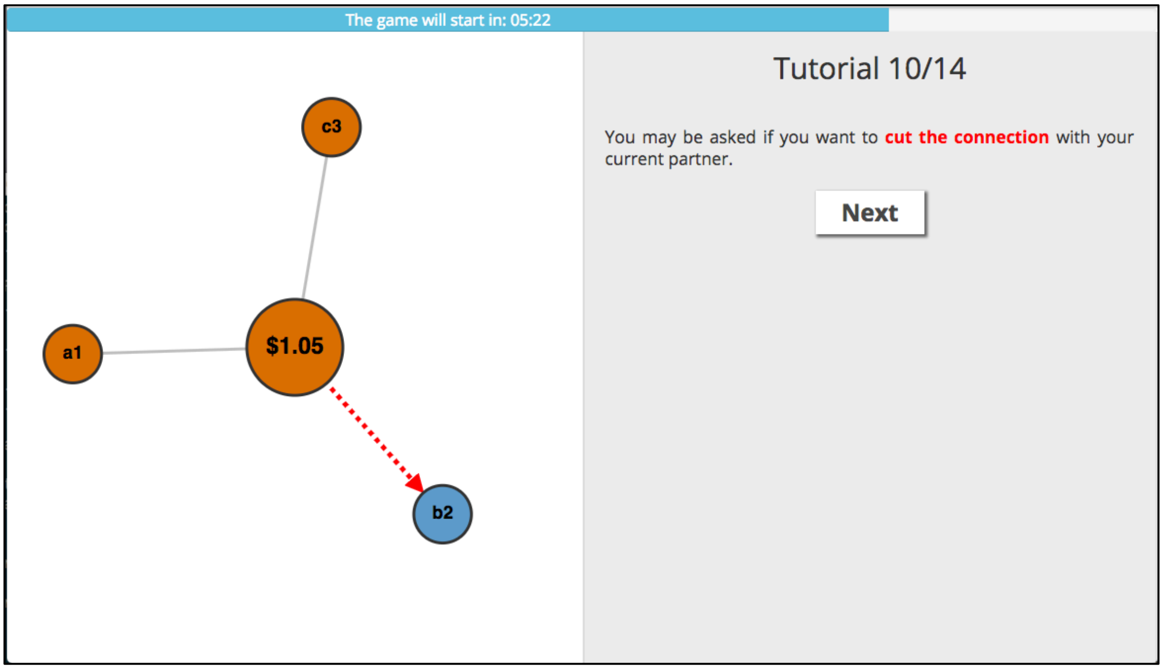

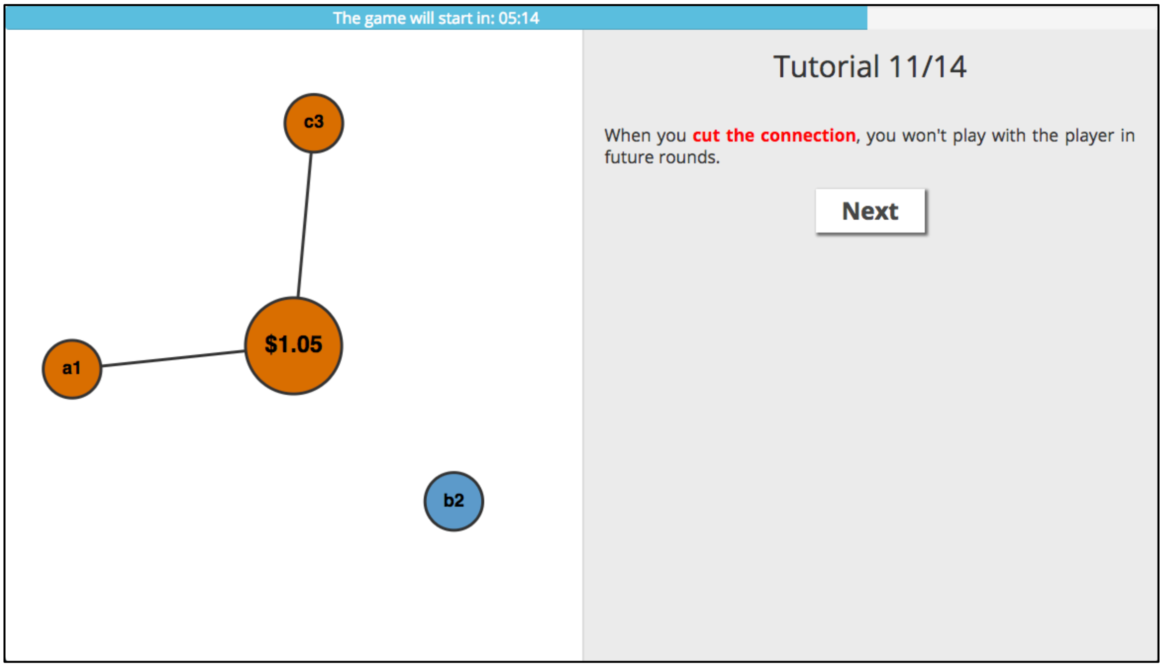

Data S1. Instruction and tutorials, Related to Figure 1. (cont.)

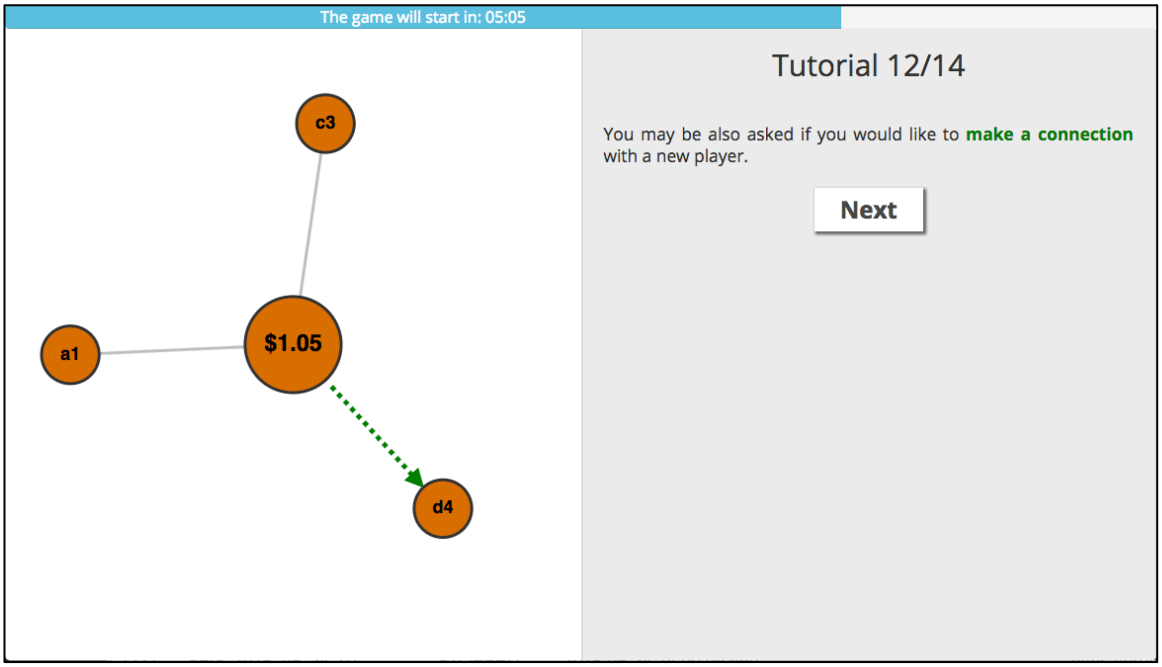

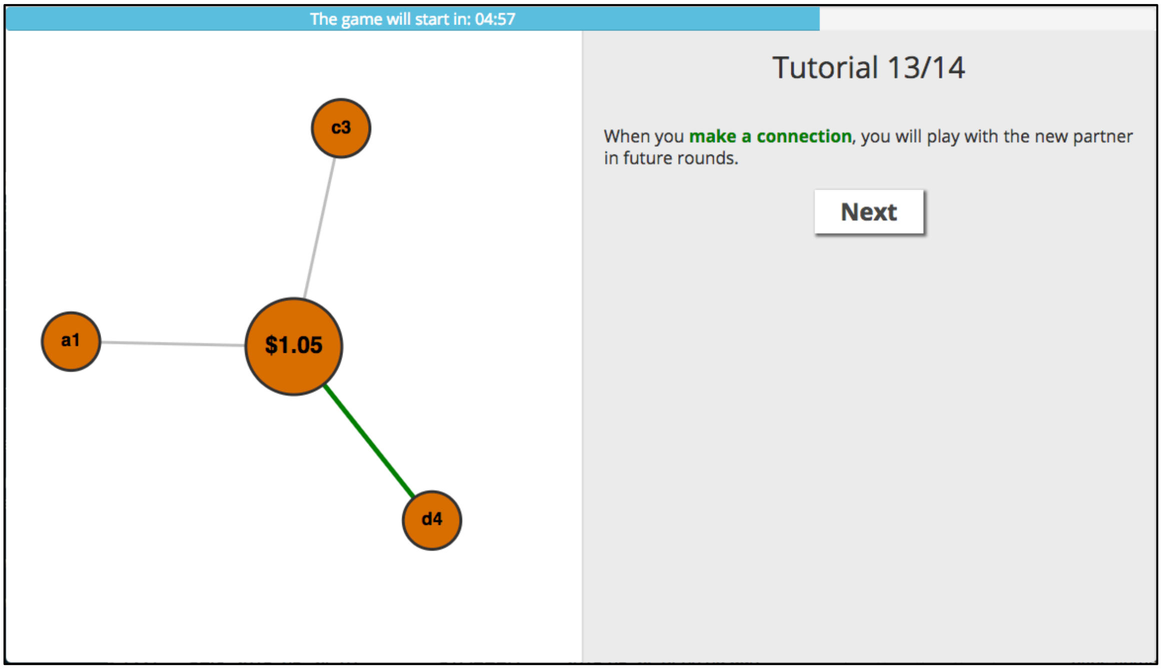

Data S1. Instruction and tutorials, Related to Figure 1. (cont.)

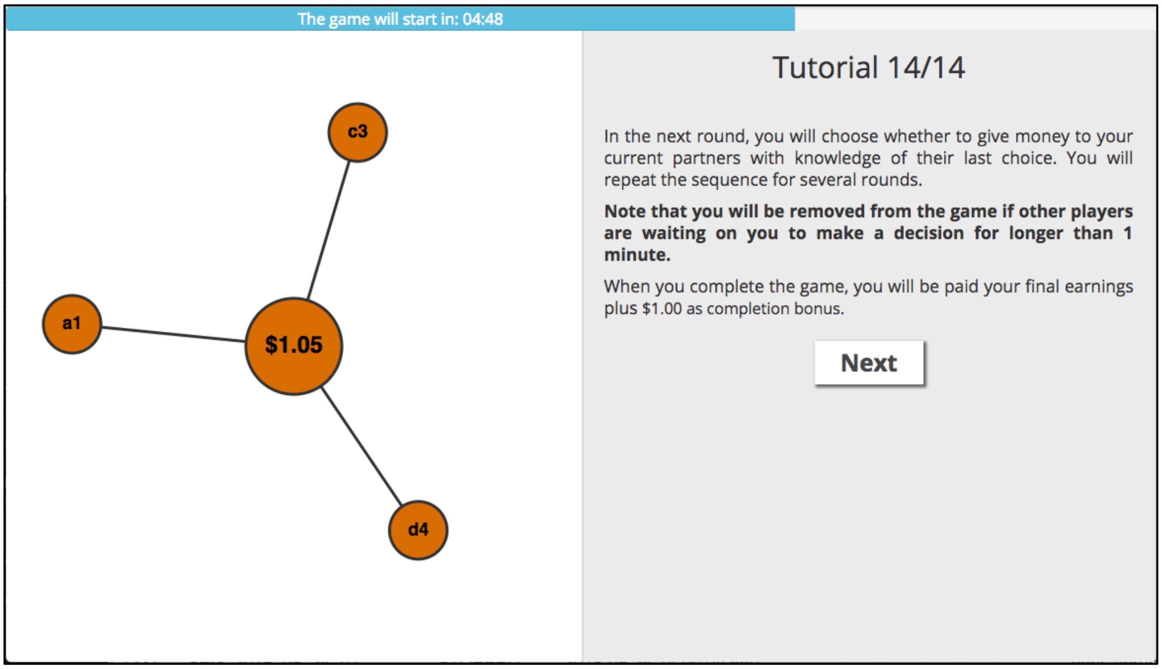

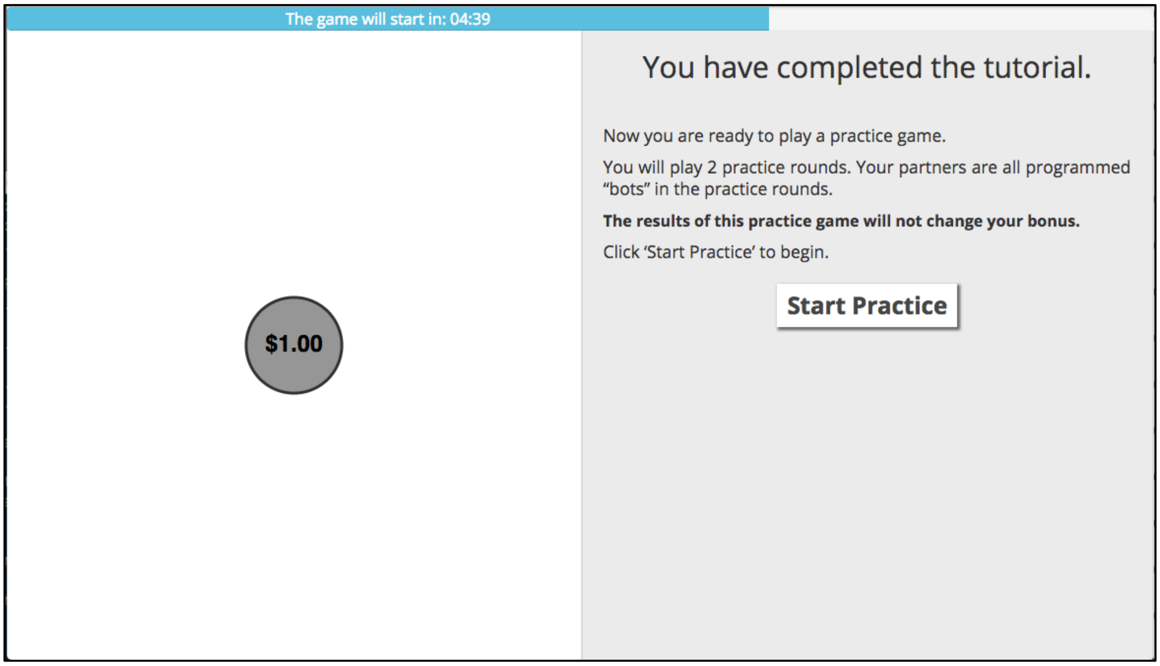

Data S1. Instruction and tutorials, Related to Figure 1. (cont.)

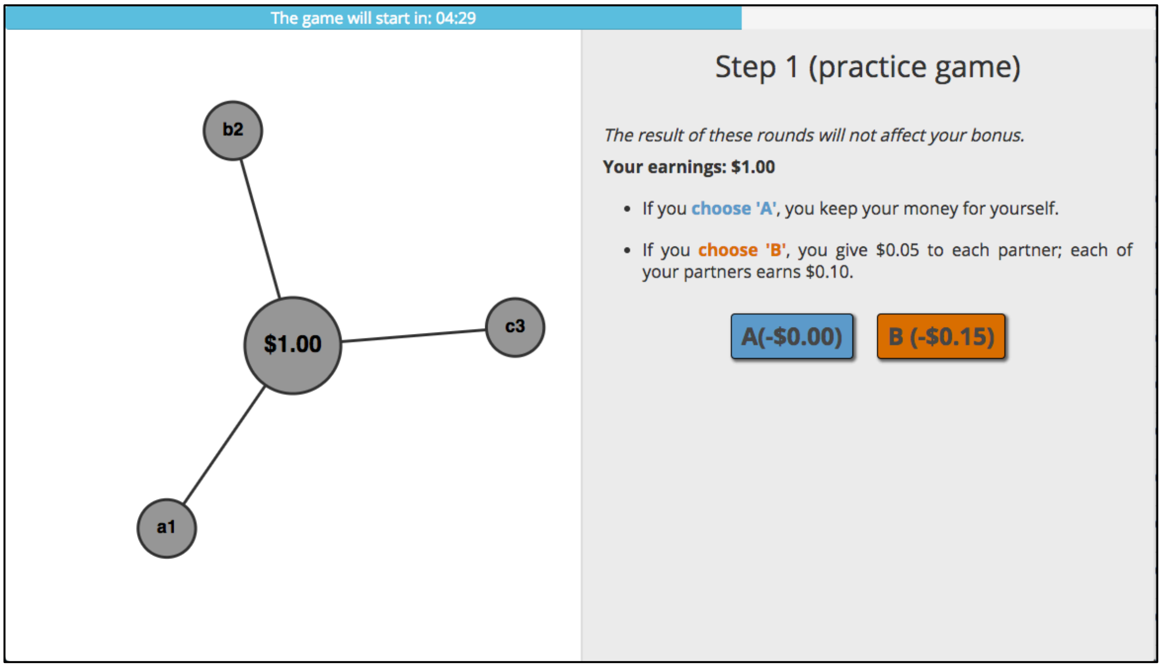

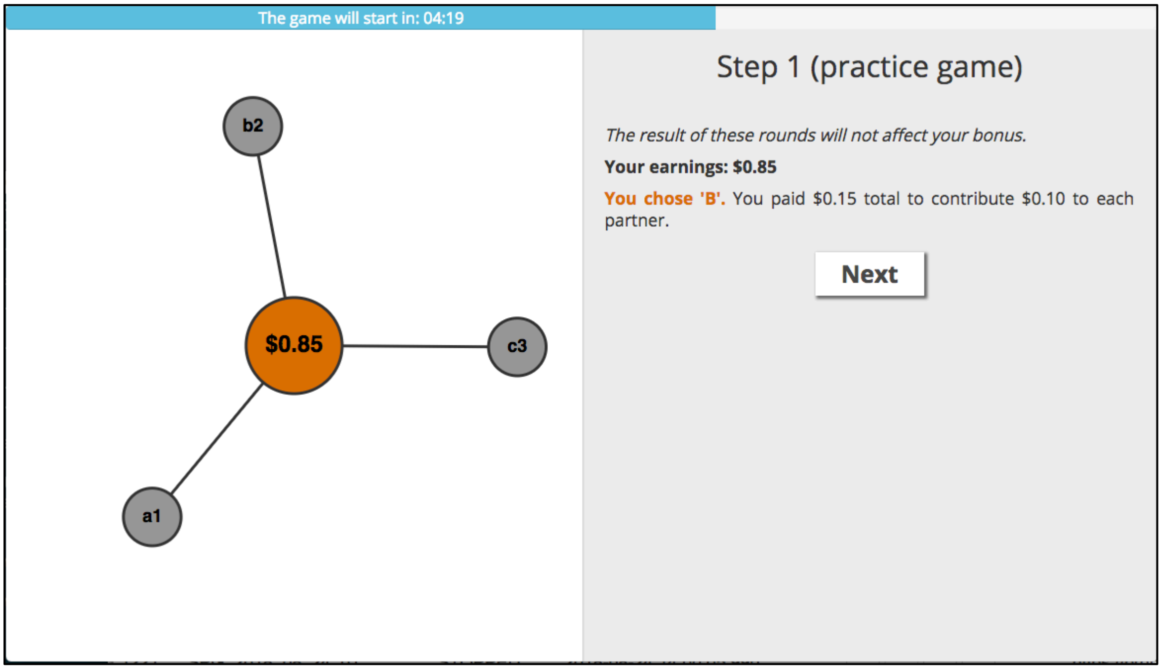

Data S1. Instruction and tutorials, Related to Figure 1. (cont.)

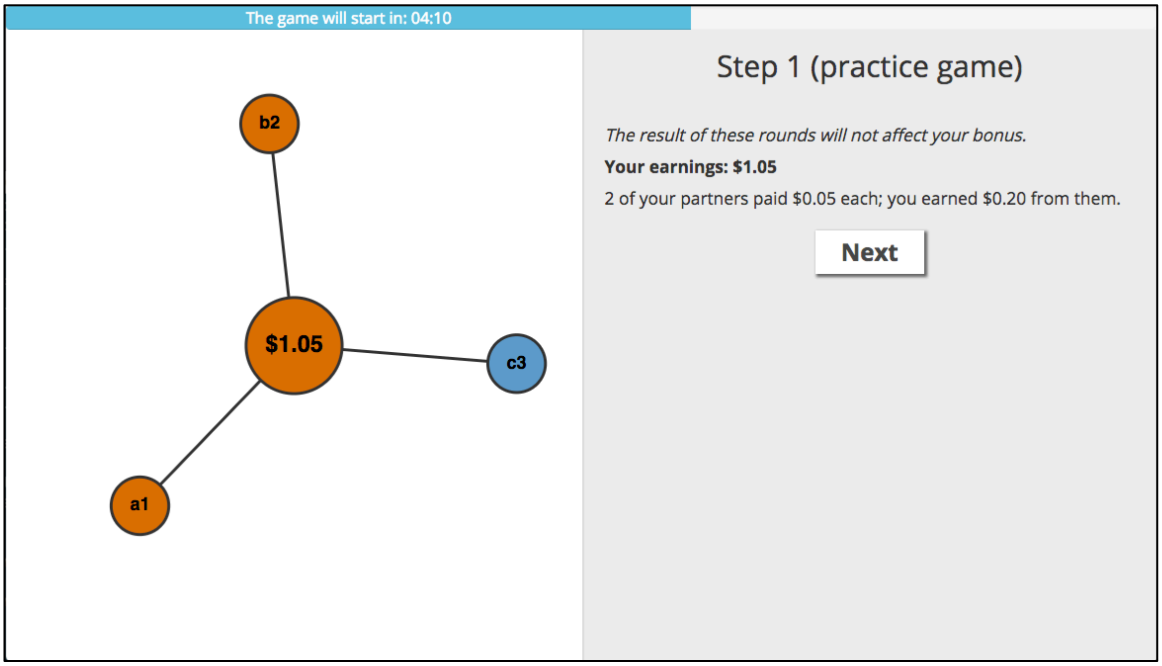

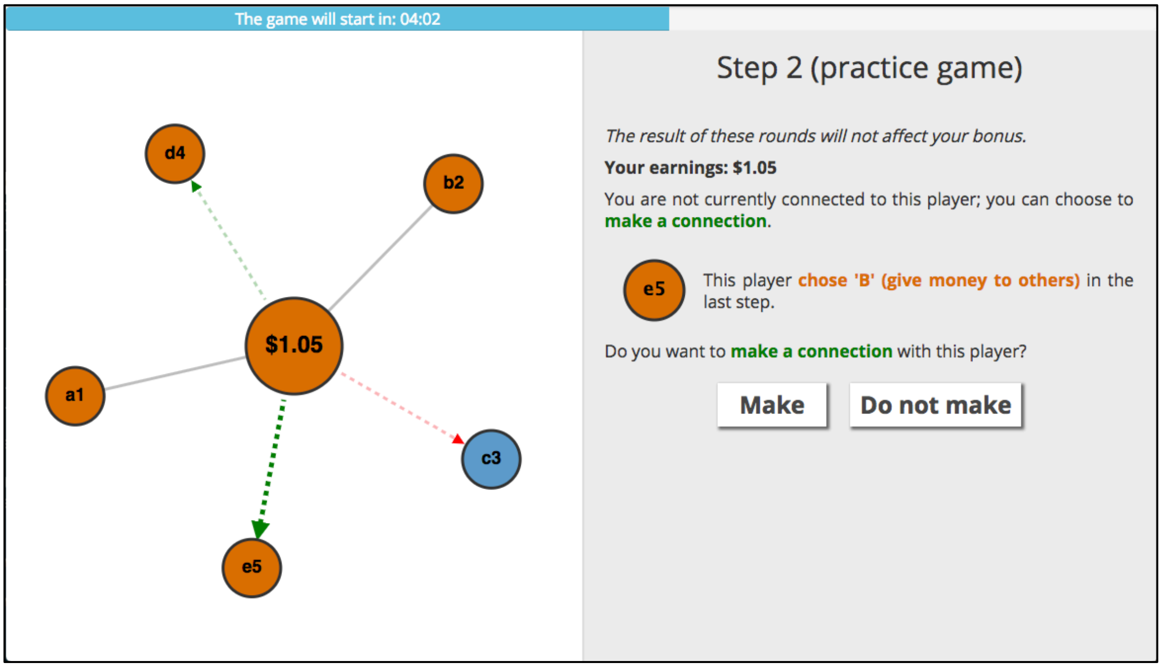

Data S1. Instruction and tutorials, Related to Figure 1. (cont.)

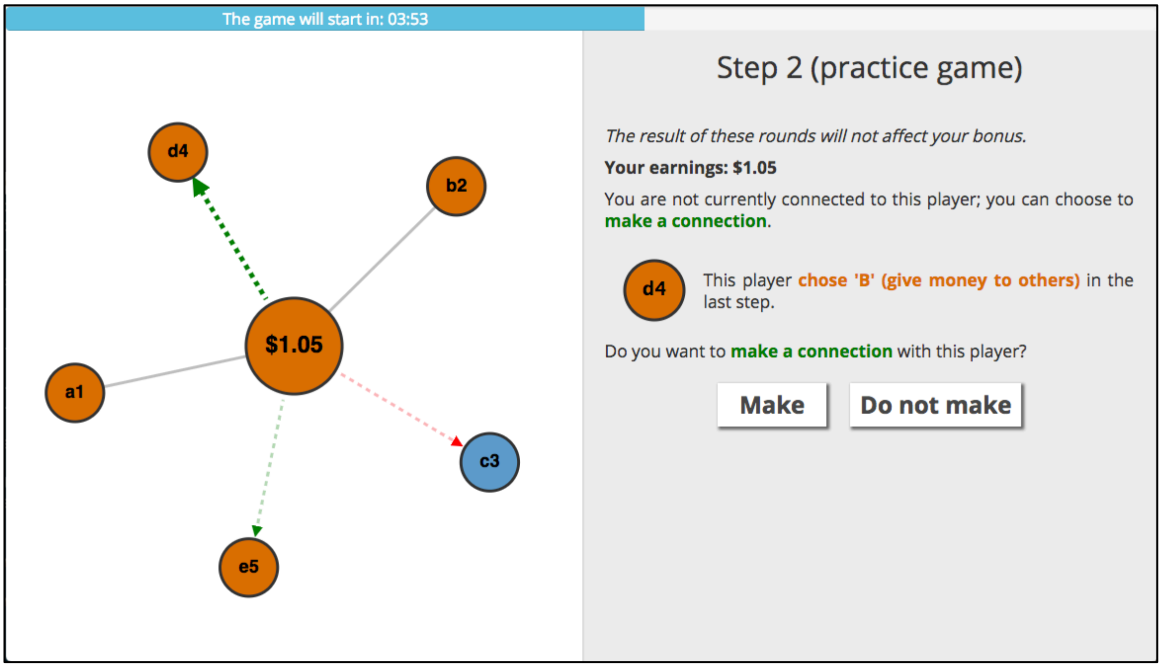

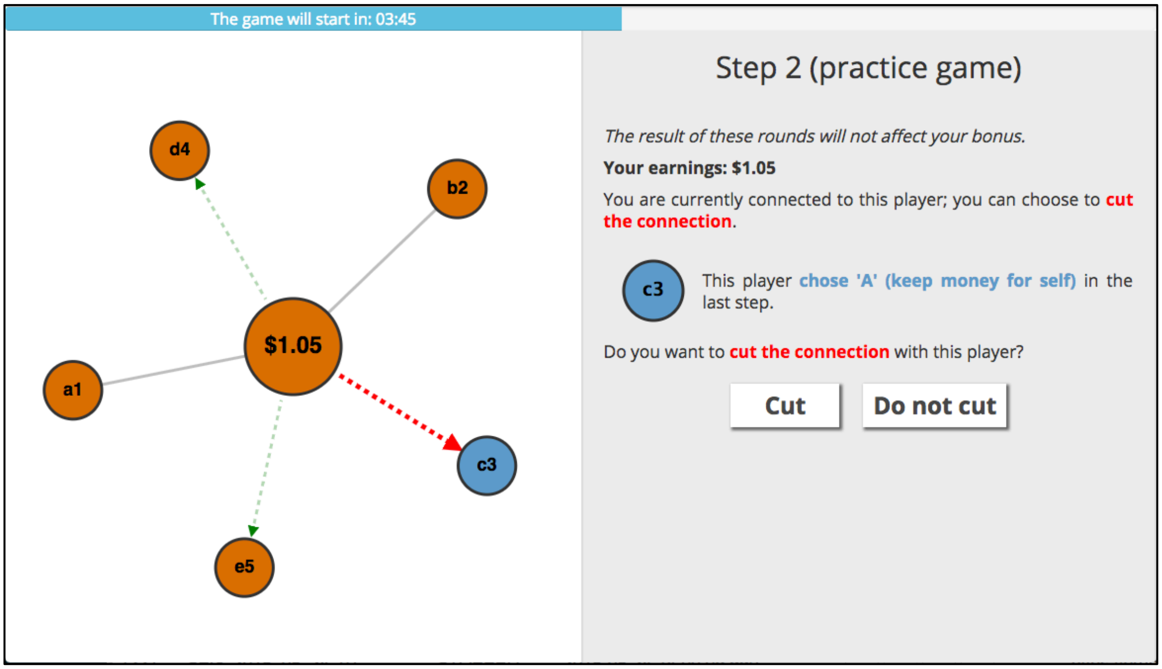

Data S1. Instruction and tutorials, Related to Figure 1. (cont.)

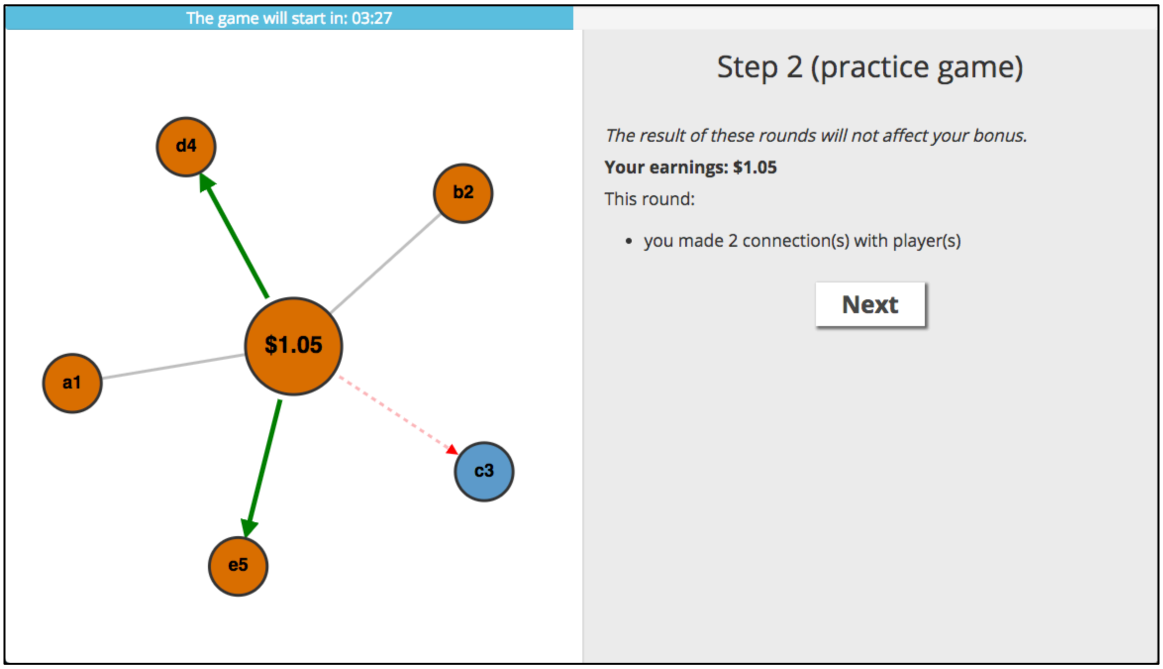

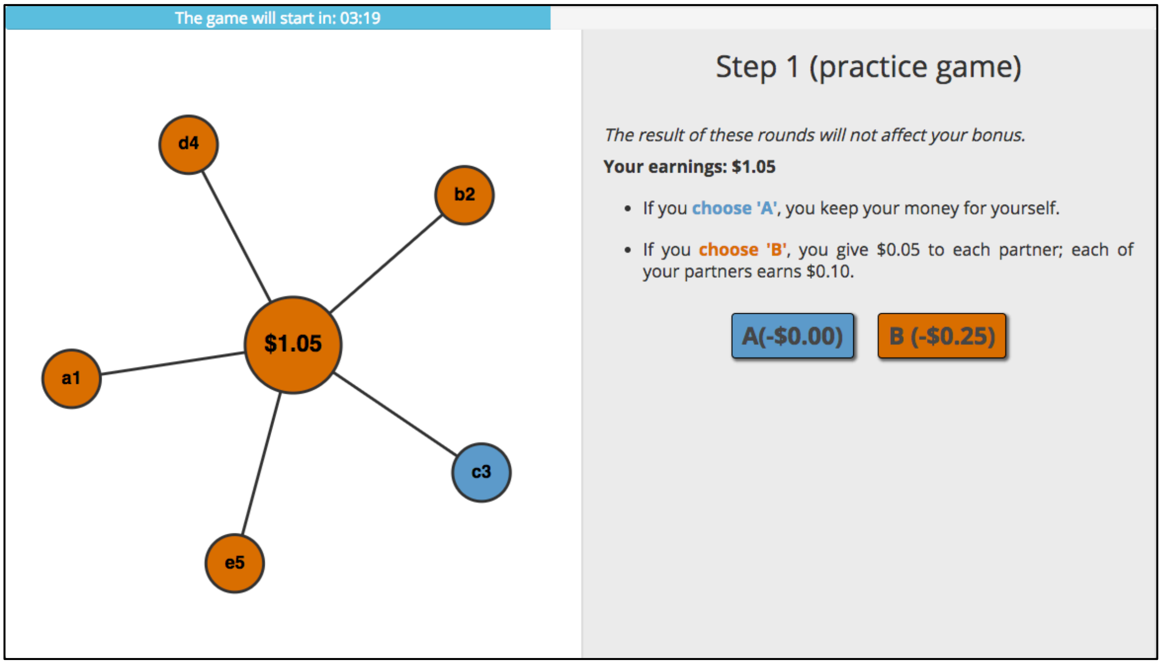

Data S1. Instruction and tutorials, Related to Figure 1. (cont.)

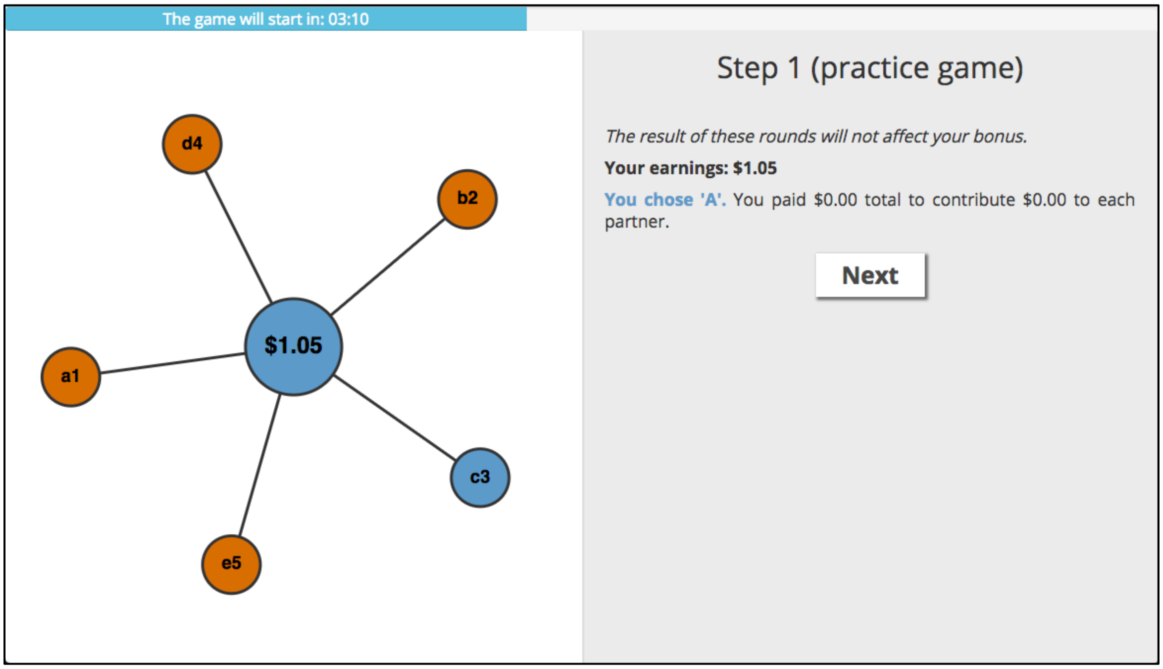

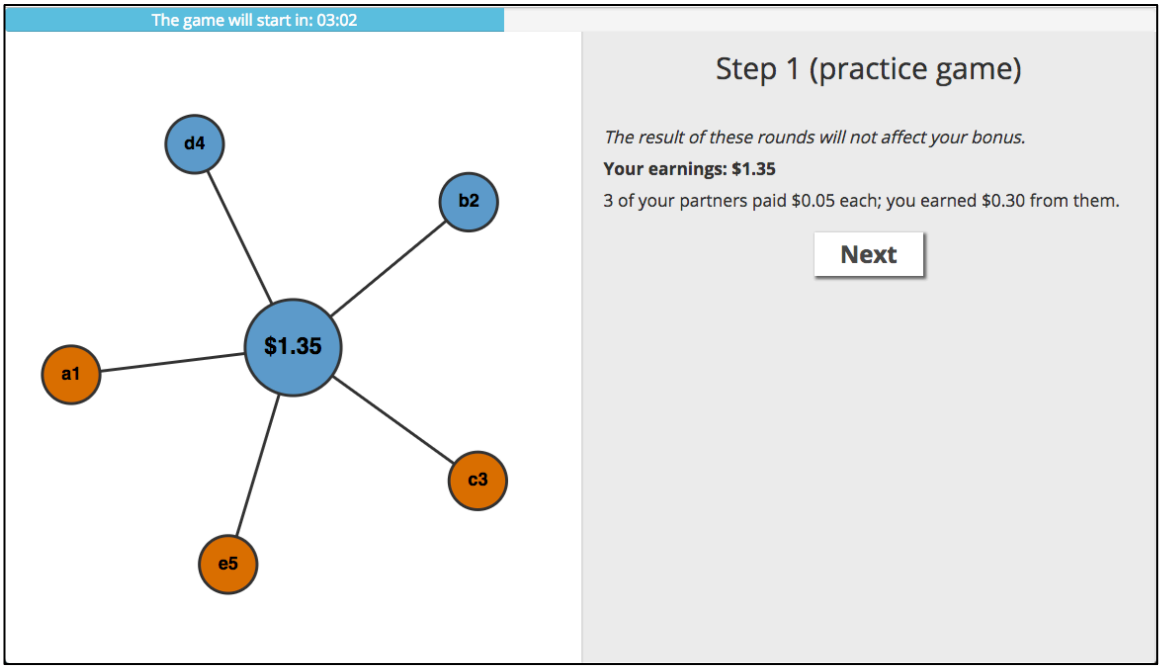

Data S1. Instruction and tutorials, Related to Figure 1. (cont.)

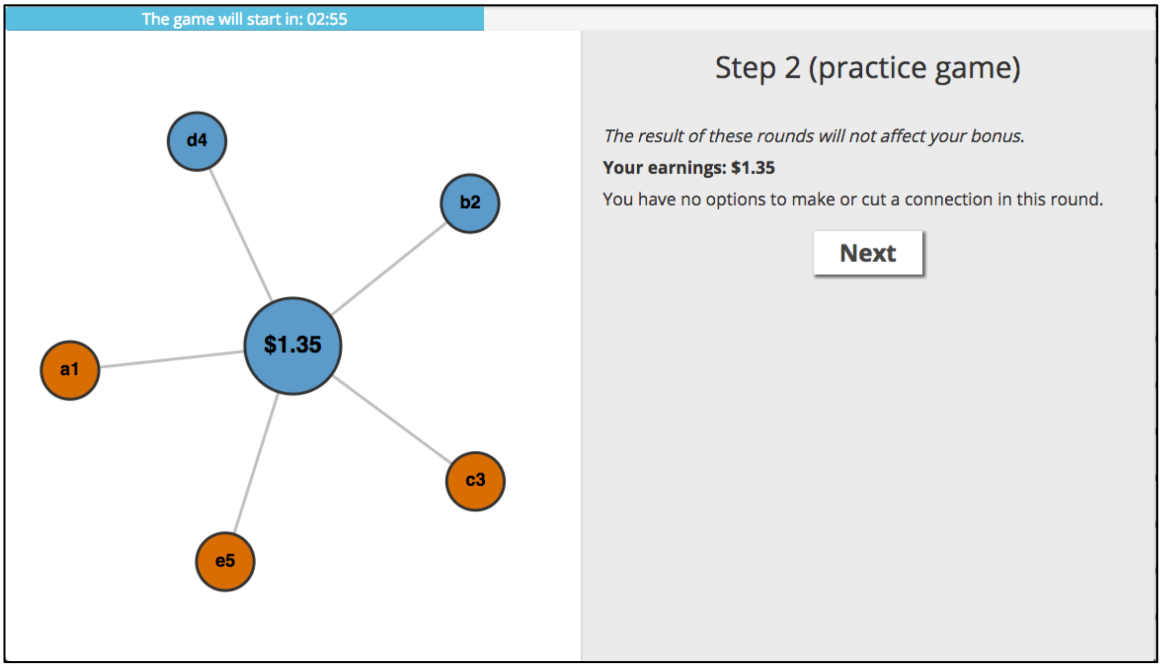

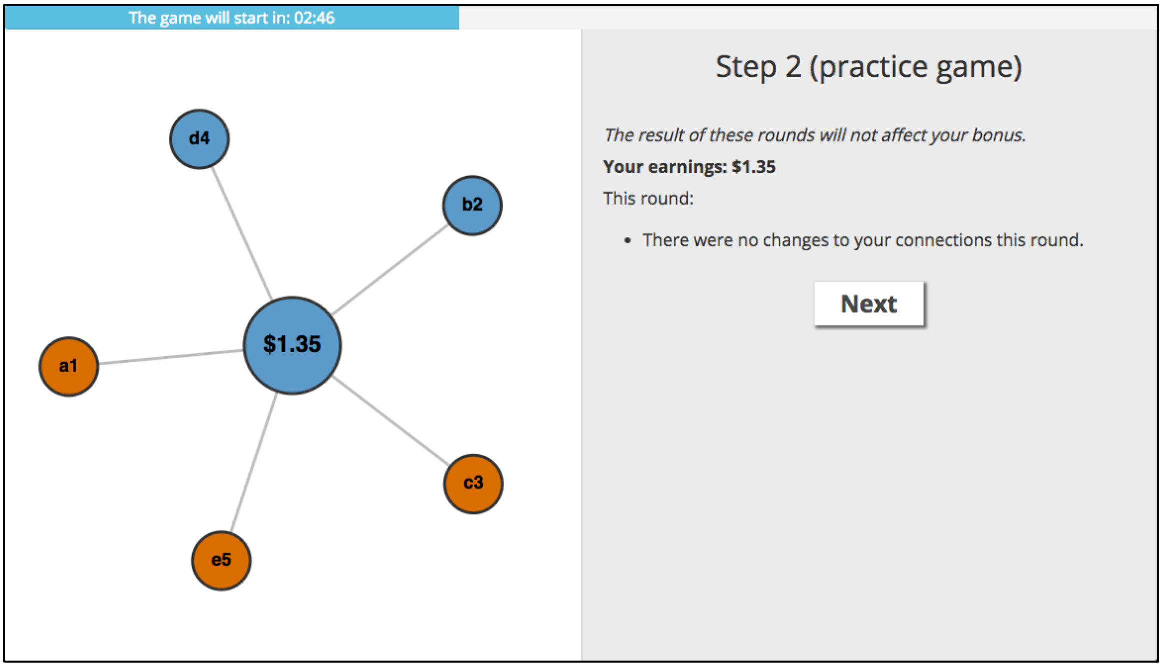

Data S1. Instruction and tutorials, Related to Figure 1. (cont.)

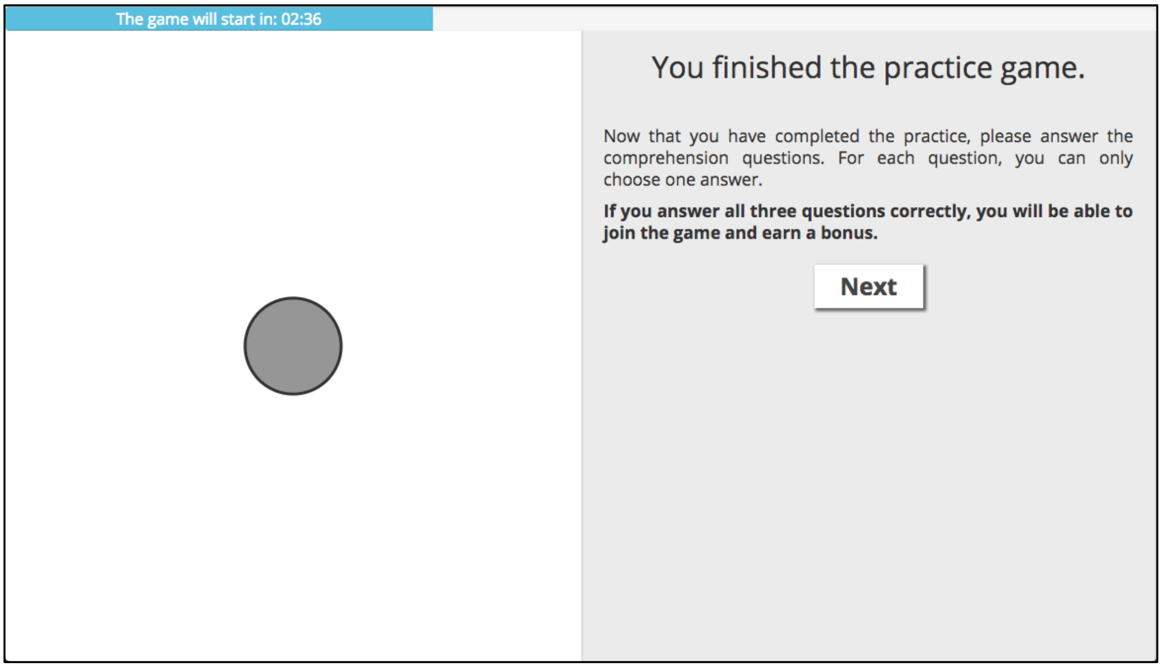

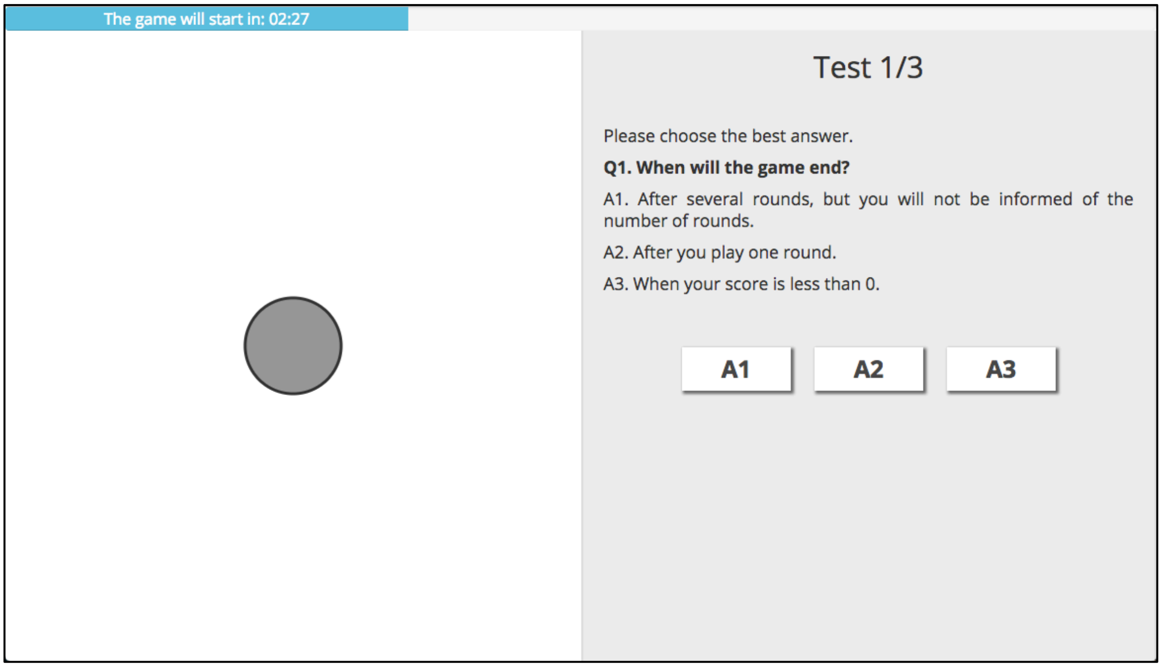

Data S1. Instruction and tutorials, Related to Figure 1. (cont.)

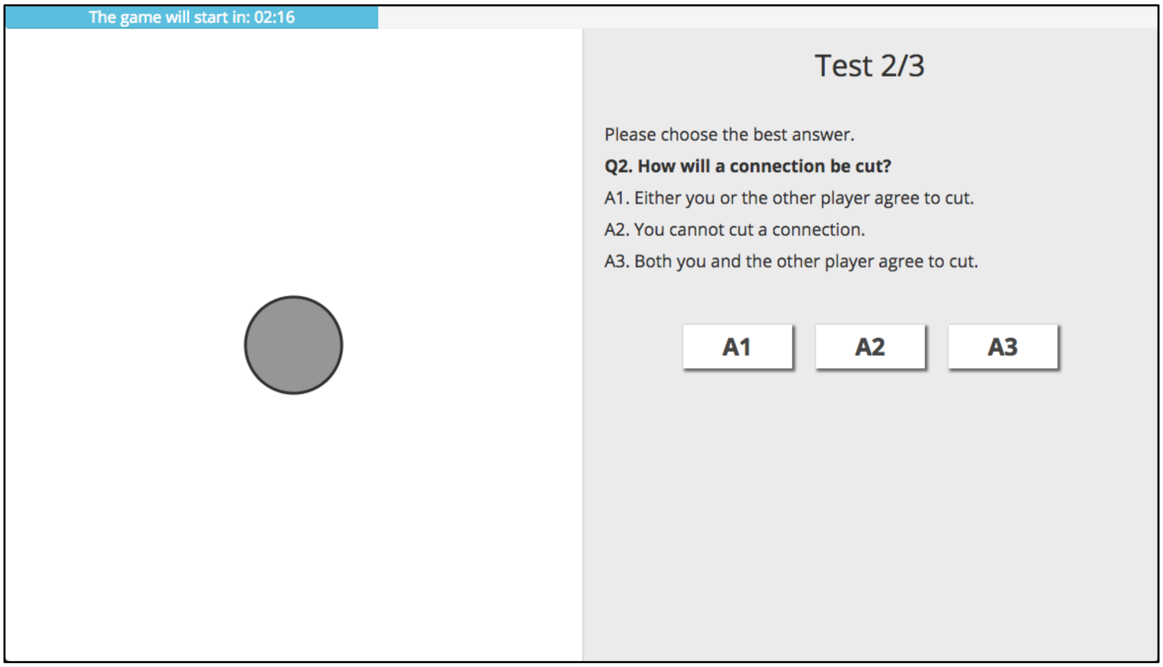

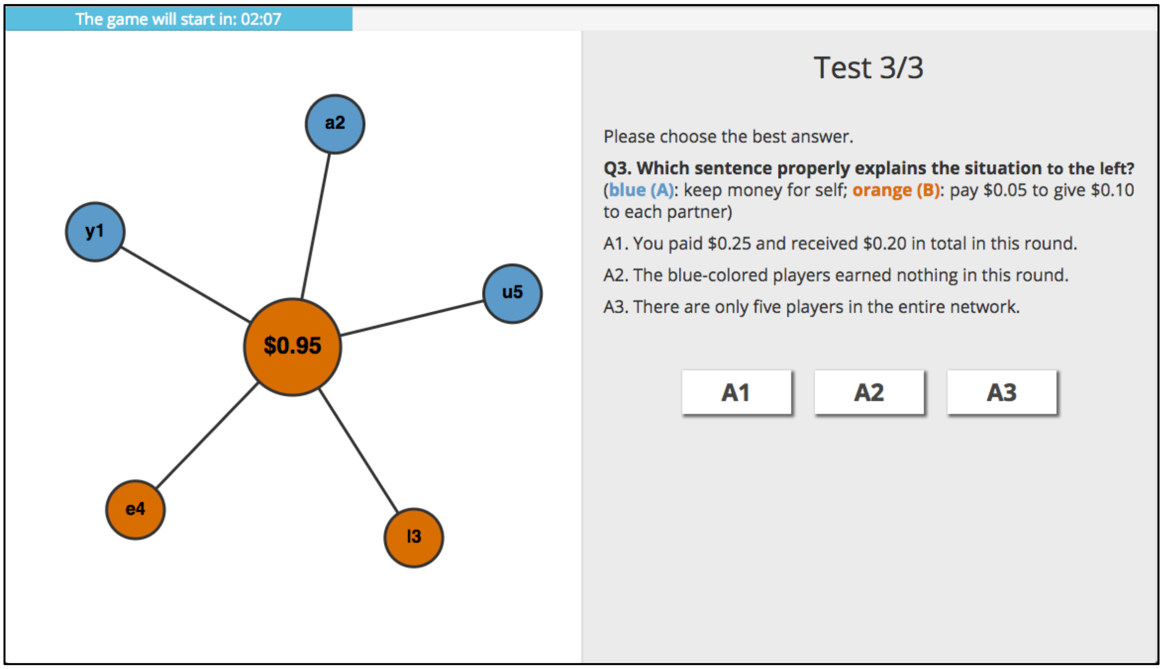

Data S1. Instruction and tutorials, Related to Figure 1. (cont.)

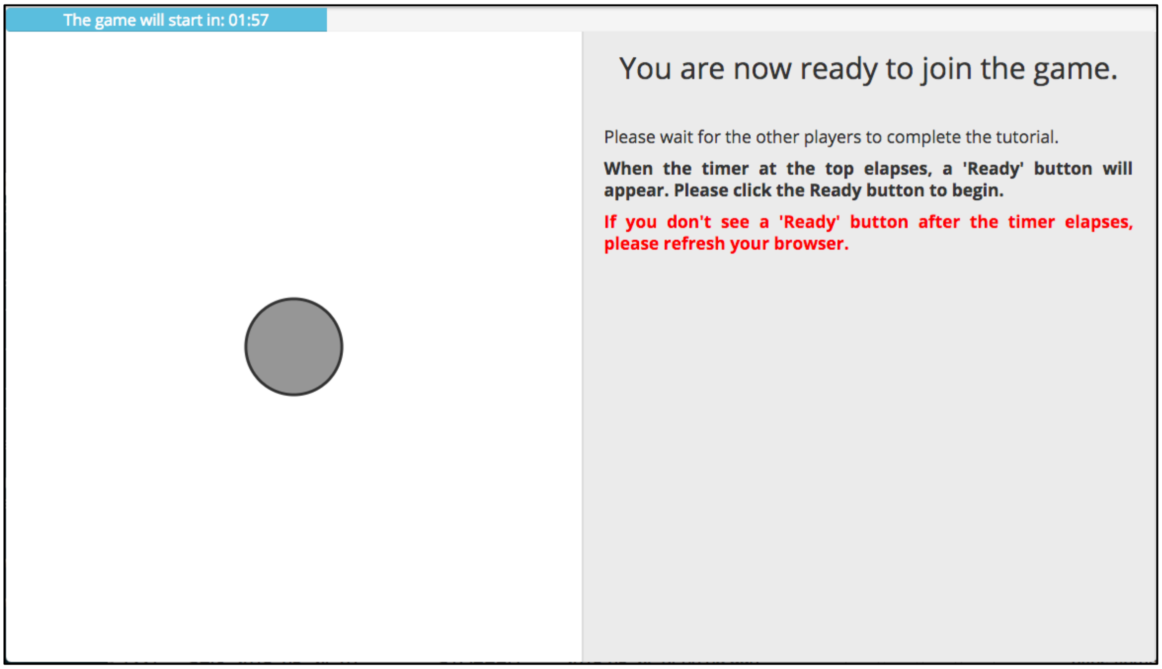

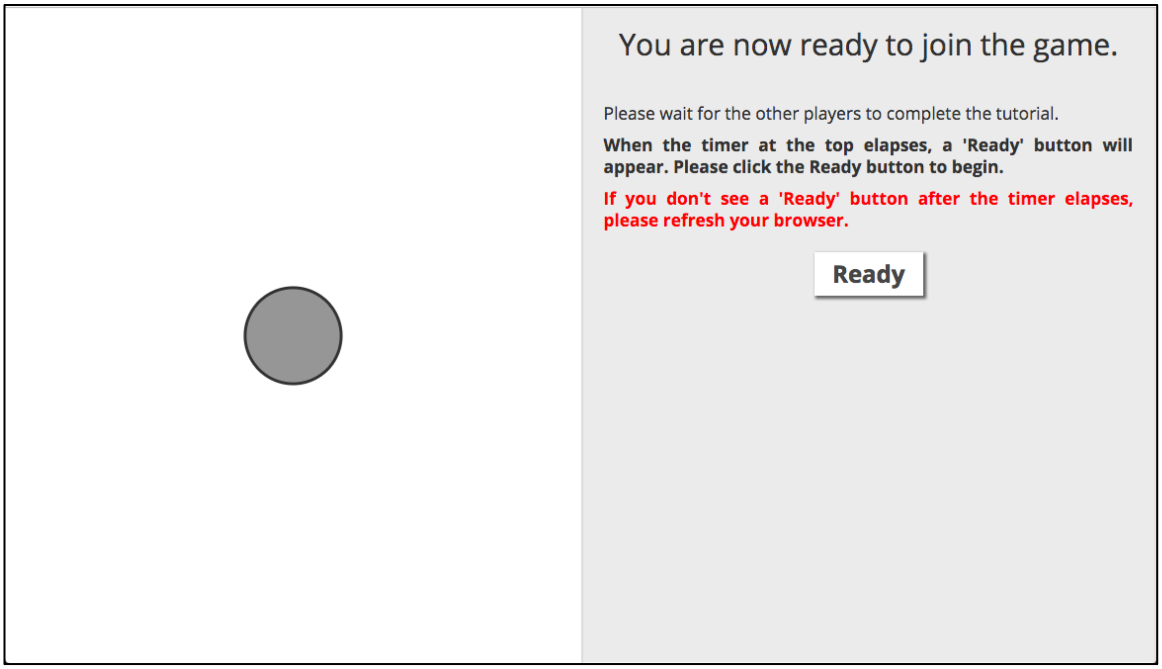

Data S1. Instruction and tutorials, Related to Figure 1. (cont.)

Sample screenshots of the real games (main experiments; bot-invisible condition):

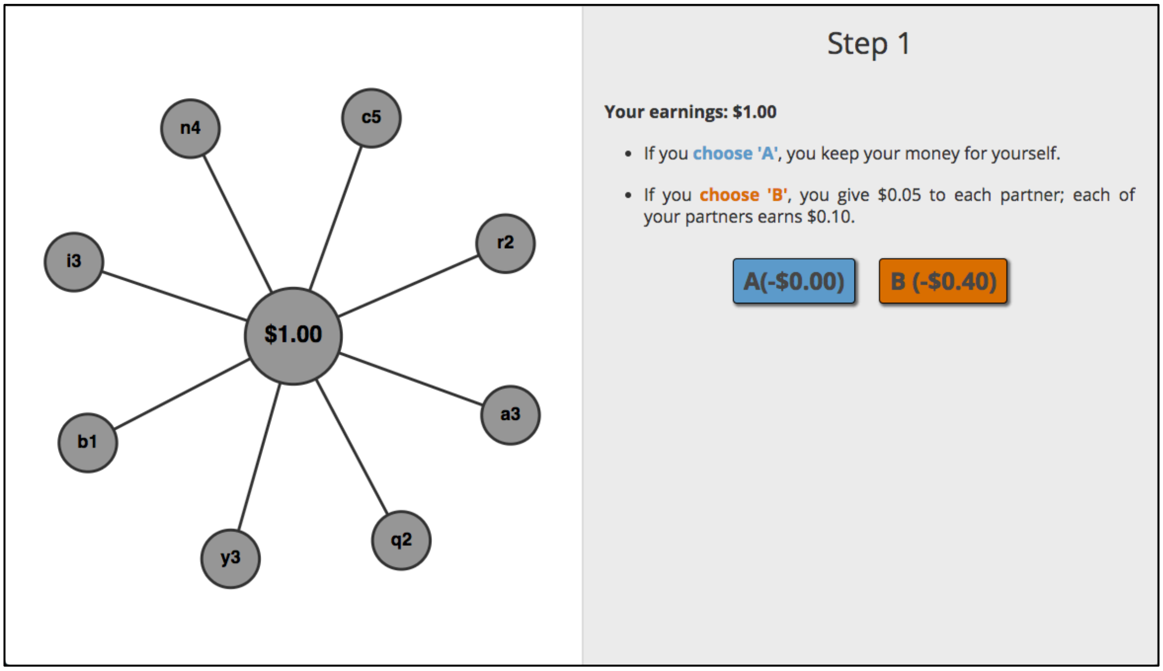

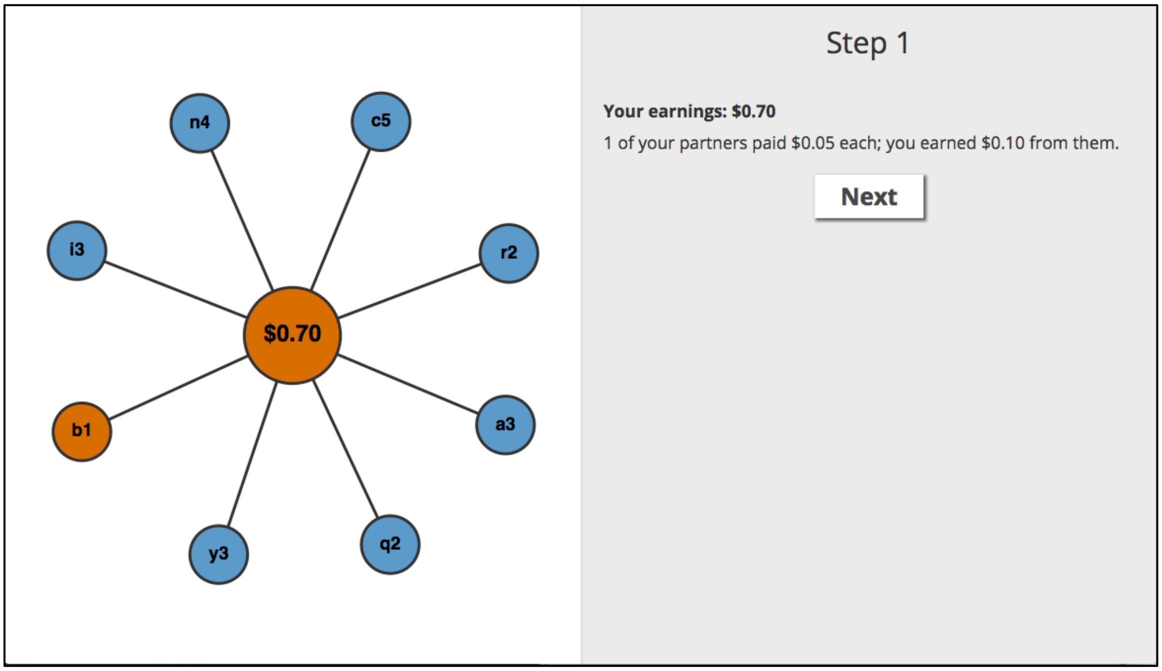

Data S1. Instruction and tutorials, Related to Figure 1. (cont.)

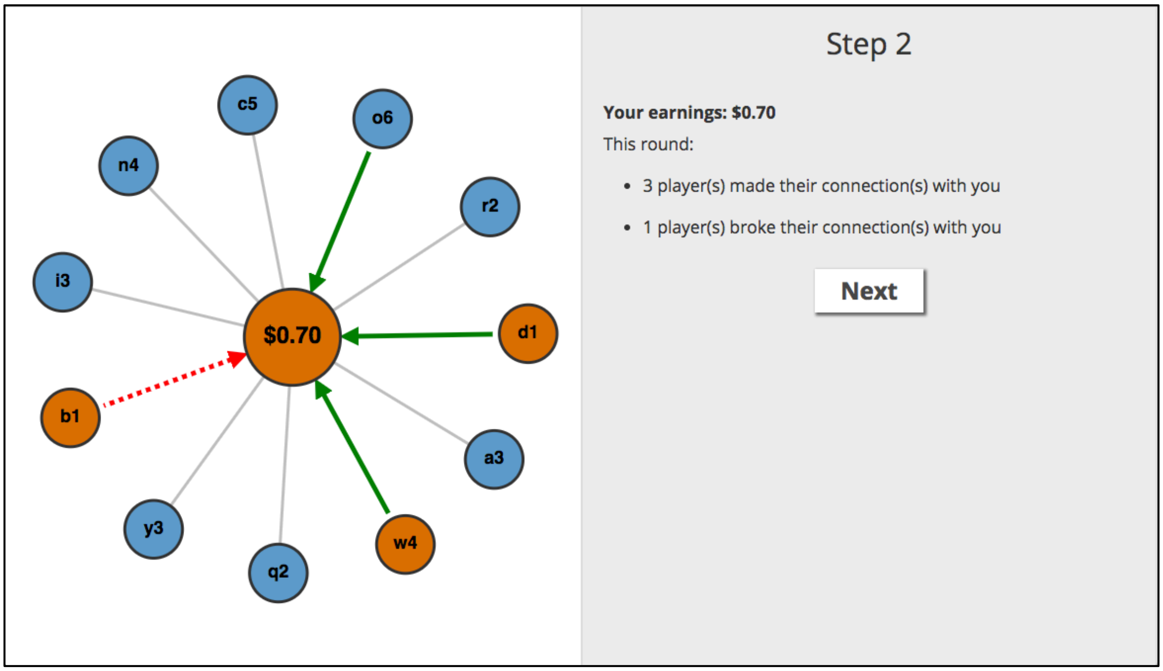

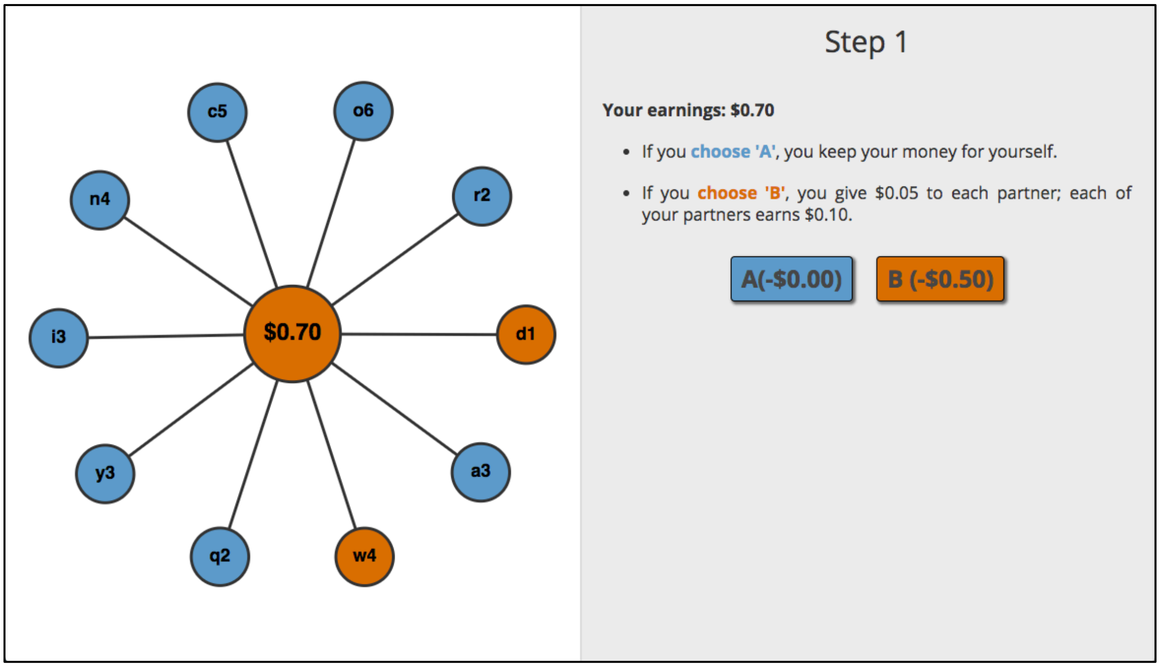

Data S1. Instruction and tutorials, Related to Figure 1. (cont.)

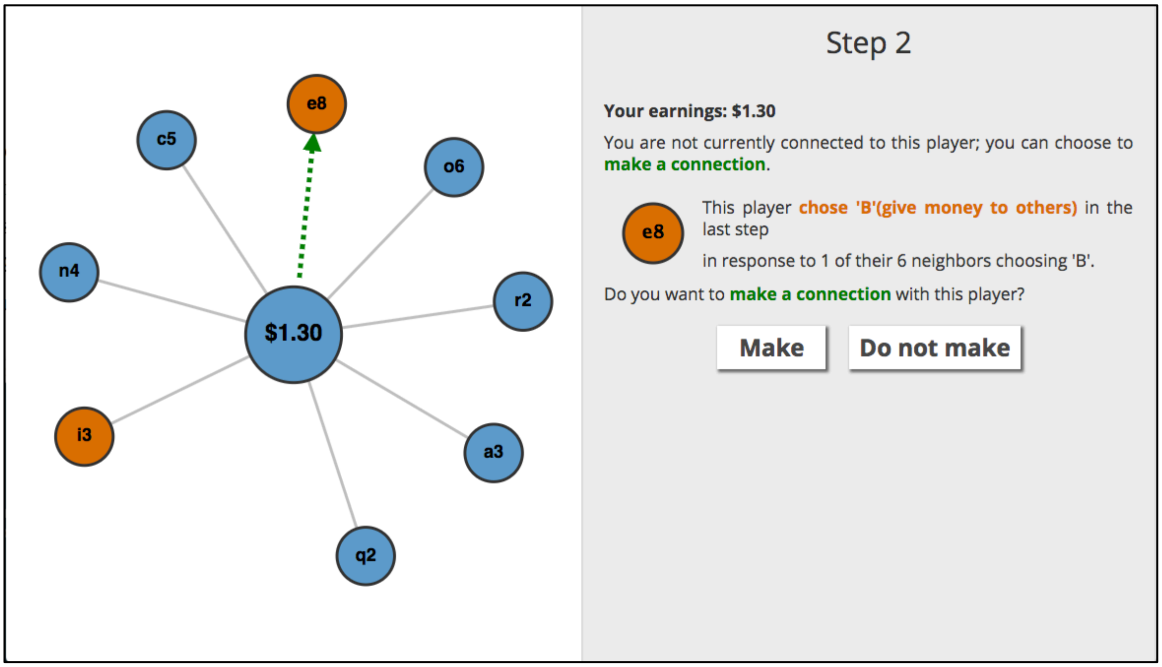

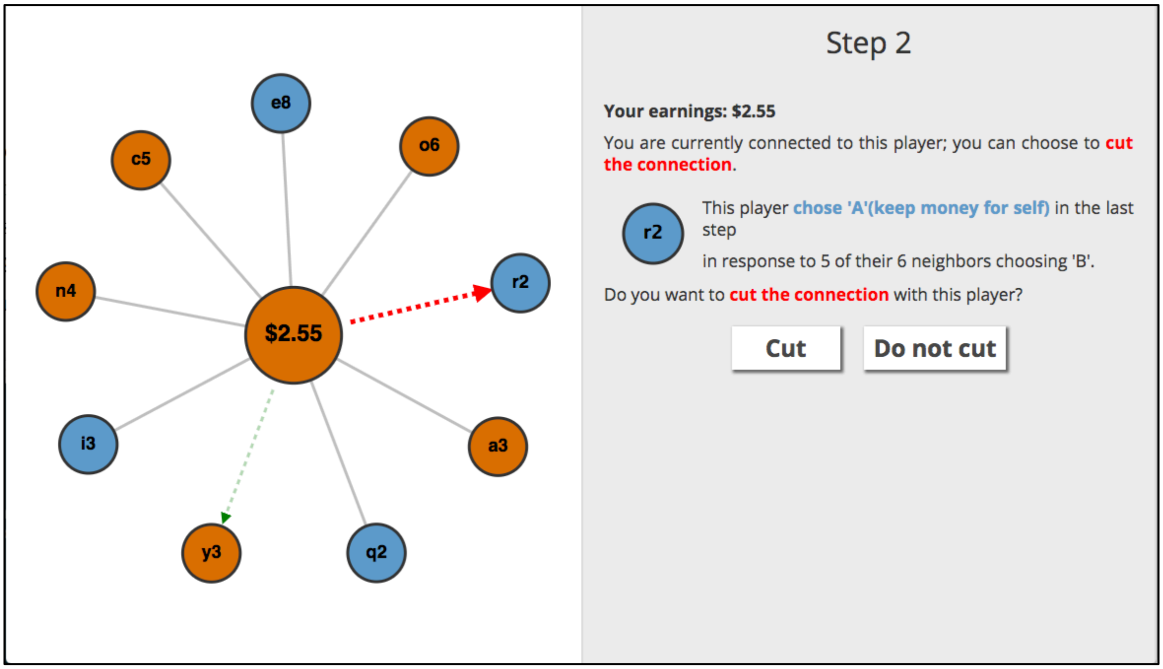

Data S1. Instruction and tutorials, Related to Figure 1. (cont.)

A session finished after players repeated the cooperation and rewiring steps 30 times.

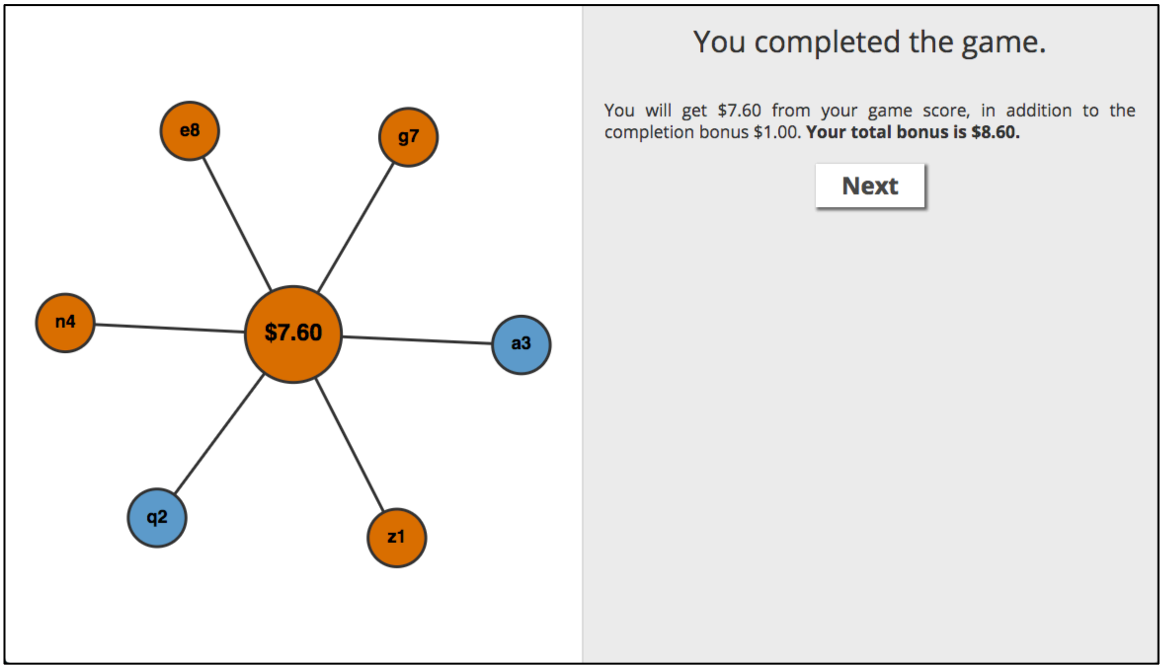

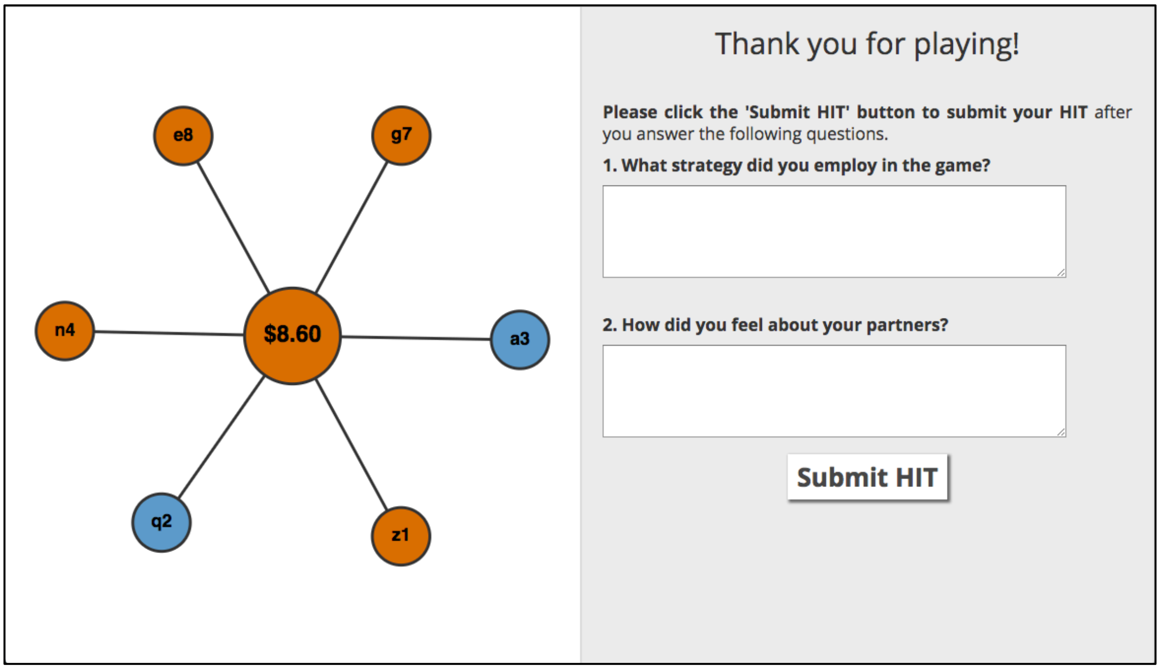

Data S1. Instruction and tutorials, Related to Figure 1. (cont.)

Sample screenshots of the real games (supplementary experiments; bot-visible condition):

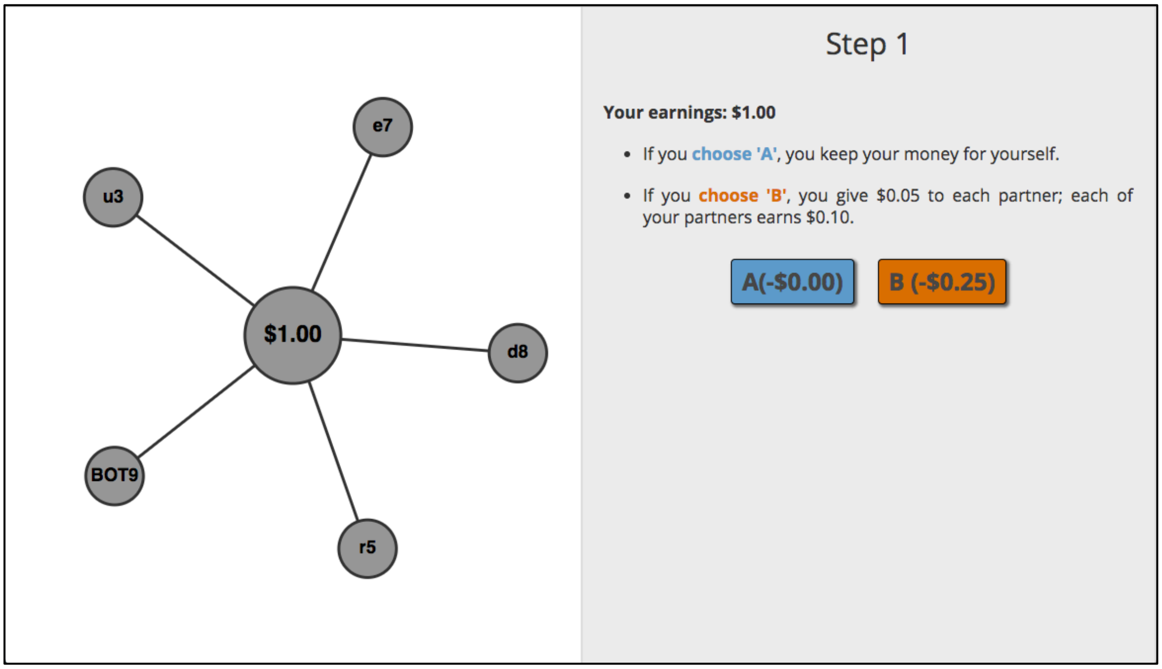

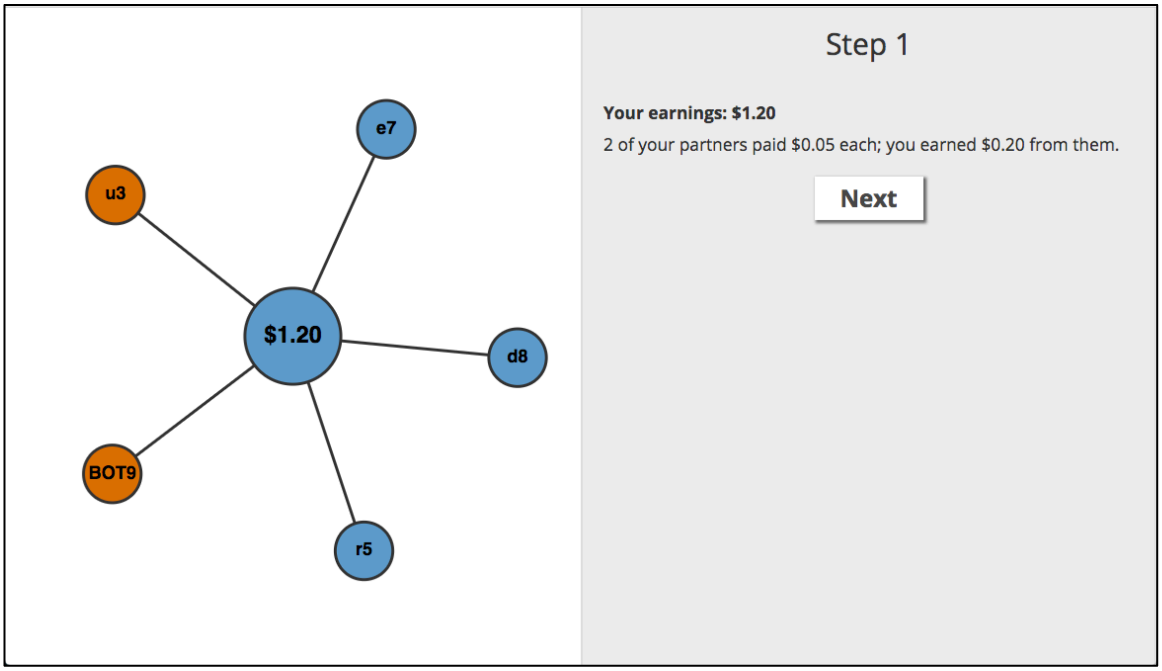

Data S1. Instruction and tutorials, Related to Figure 1. (cont.)

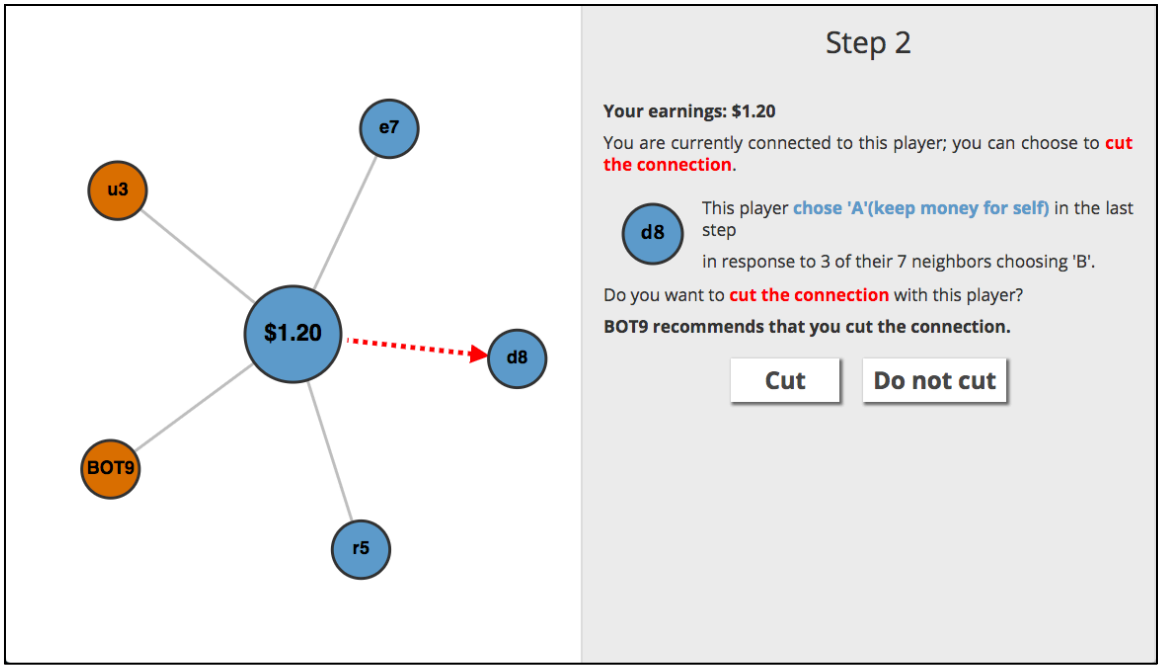

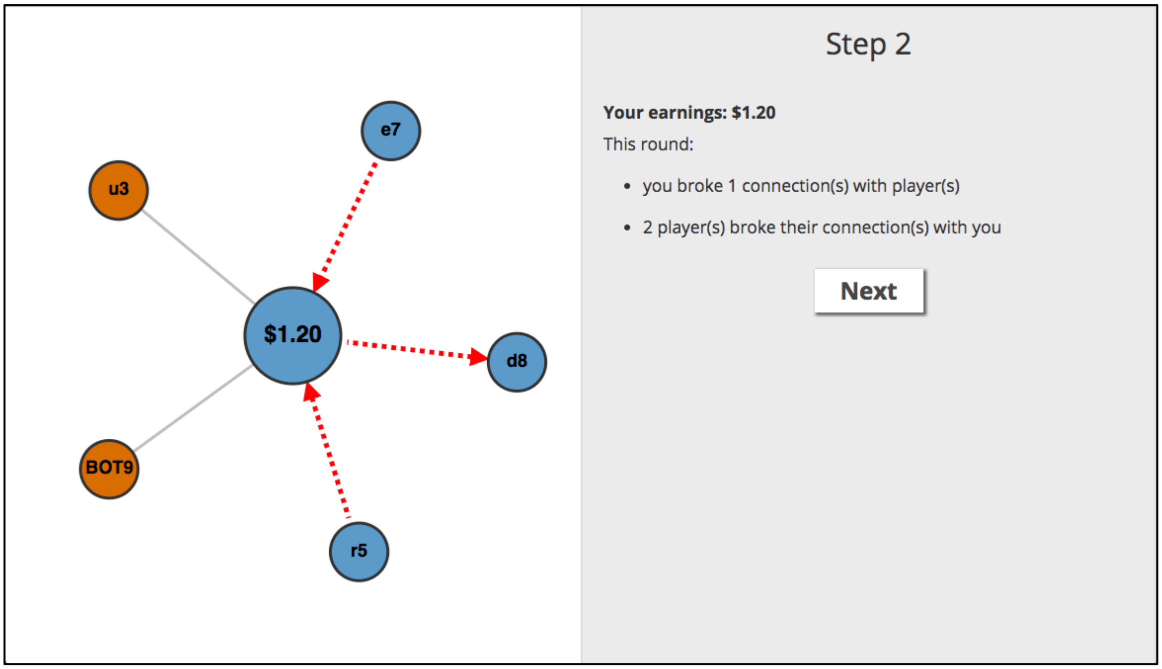

Data S1. Instruction and tutorials, Related to Figure 1. (cont.)

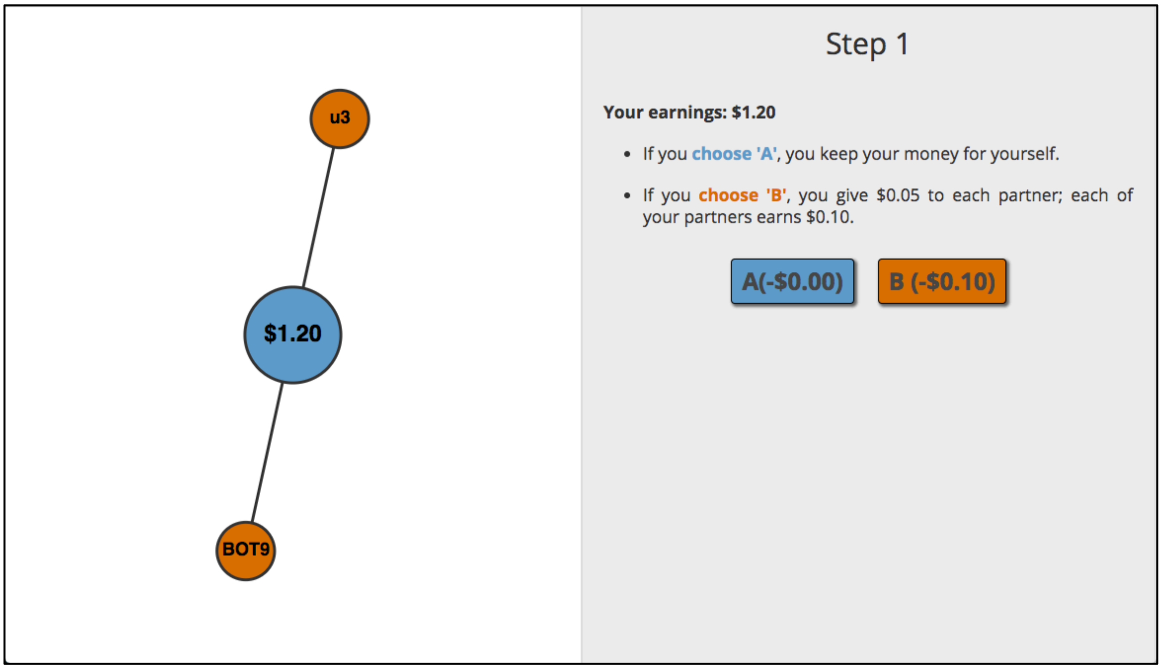

2. Transparent methods

Recruitment procedure

A total of 1,024 subjects ($N_{subject}$=896 for Experiment 1; $N_{subject}$=128 for Experiment 2) participated in our incentivized decision-making game experiments, always in groups of 16 people ($N_{session}$=64 sessions). Subjects were recruited using Amazon Mechanical Turk (MTurk) via our breadboard software platform (which we have made available at breadboard.yale.edu). MTurk is an online labor market in which employers contract with workers to complete short tasks for relatively small amounts of money. Many studies have demonstrated the validity of behavioral experiment data gathered using MTurk (Rand, 2012; Thomas and Clifford, 2017). Behaviors of MTurk subjects in stylized economic games are correlated with their actual behaviors in a real-world situation (Peysakhovich et al., 2014). MTurk subjects often receive a baseline payment, plus an additional bonus depending on their performance. For this study, subjects received $2.00 when they completed the tutorial to join a game and $1.00 when they completed the entire game. In addition, subjects also received a bonus payment based on their own earnings during the networked cooperation game, which averaged $9.50.

Experimental setup

Our participants interacted anonymously using *breadboard* and playing in a browser window. We prohibited subjects from participating in more than one session of the experiment by using the unique identifications for each subject on MTurk. All the subjects were informed about the use of their behavioral data for research purpose upon

enrollment in the experiment. The experiments were conducted from August to November 2018.

Subjects were placed in a group of humans with a size of 16 and arranged in a social network with an Erdős-Rényi random graph configuration in which 30% of ties were present, on average. In a bot-integrated session of Experiment 1, each subject additionally received one connection with a bot. Subjects were therefore initially connected to an average 4.41 (s.d. = 1.76) human neighbors and 1 bot neighbor (i.e., average 5.41 neighbors in total). In Experiment 2, we created a random network with 16 human subjects, as above, and then added 1 bot having 5 ties to the network, which resulted in an average of 5.13 neighbors in the whole group at the onset (sometimes including the bot). Subjects could identify each neighbor by a name label that was randomly generated with a number and a letter (such as "j2" and "y9"; see Data S1).

The subjects played a Public Goods game lasting 30 rounds with their network neighbors (which consisted of other human subjects and possibly bots). At the beginning of the game, human subjects received $1.00 as their initial endowment. In each round, all the subjects chose whether to cooperate, by reducing their own endowment $0.05 per neighbor in order to increase the endowment of all neighbors by $0.10 each, or to defect, by paying no cost and providing no benefits. Subjects made the same choice with all their neighbors. When a subject did not have any neighbors in this step, the subject was not given the cooperation choice at the round.

After making their cooperation choice, subjects were informed of the choices made by their neighbors. Then, subjects sometimes had the opportunity to change their neighbors by making or breaking ties ("tie-rewiring" options). Specifically, 5% of all pairs of human subjects were chosen at random in each round and given the opportunity to rewire their networks. If a tie already existed between the two subjects, then one of the two was picked at random to be allowed to choose whether to voluntarily break the tie with the other; if a tie did not already exist between the two, a randomly selected subject from the pair was given the option to form a tie. When making this decision, subjects were aware of whether the person to whom them might disconnect or connect had cooperated or defected in the past round. In addition, they were also informed how many total neighbors and cooperative ones the focal person had in his or her immediate environment.

At any point during the game, if a subject was inactive for 15 seconds, the subject was warned about being dropped. If they still remained inactive after 15 seconds, they were dropped. Since dropping subjects changed the network structure, we calculated all the network metrics in each session of 30 rounds and used the average for each subject. The dropped subjects were prohibited from joining another session of this experiment.

Within this basic setup, we introduced 16 bots into the network of 16 human subjects (except for the control sessions without bots) in Experiment 1. Each bot had only one tie and connected with a different subject (that is, each subject had a bot among their neighbors) at the beginning of a game. We used the artifice of single-tie bots so as to fix

the amount of intervention across sessions and treatments to 16 total ties with bots at all times and in all treatments. These single-link bots keep the same amount of intervention (in terms of ties and money) across sessions and treatments. Moreover, this set-up made all the subjects interact with one cooperative (or Tit-for-Tat) bot over rounds. Thus, we can be sure that whether cooperation collapses is unrelated to any heterogeneity of bot influence across groups. If only some of the subjects had bots at the outset, the ineffectiveness (or effectiveness) could come from the possibly biased characteristics of subjects that bots attached to by chance. But here, since every subject equally has one connection with a bot, this is not an issue.

Bots always chose cooperation in the game (except for the sessions of Tit-for-Tat bots); that is, they gave the same amount of cooperation benefit into a social system ($1.60 = 16 × $0.10 per round) to the subjects who connected with them. Bots never connected with each other.

Subjects were not informed that there were bots in the game (except in the extra condition of bot visibility). In their local view, subjects could identify every bot as a neighbor in the same manner as other subjects using the name labeling system.

In Experiment 2, we explored the possibility of a minimal intervention based on the results of Experiment 1. In this experiment, we added 1 bot having 5 connections to a network of 16 human subjects. In contrast of Experiment 1, some subjects had a connection with the bot and the others did not. Like Experiment 1, bots always chose

cooperation in the game so that they gave a total of \$0.50 to the network of humans at each round (\$0.50 = 5 × \$0.10).

Statistical analysis

Analyzing the data from our experiment requires more than an analysis of the average final values across treatment groups (which leverages the randomization of the experiment). For instance, the average values by round do not represent directly the slopes of the change over a session. Moreover, ratio data represented as the cooperation rate, which is limited to be between 0 and 1, does not come from a population that is normally distributed. In addition, multiple observations from the same subject and observations from multiple subjects within the same session are not independent. Thus, we need to deal with the nested structure of errors in our statistical analysis.

Hence, we used a statistical analysis based on a GLMM involving logistic regression with nested random effects. GLMM estimates coefficients in the linear predictor and random effects, which comes from individual differences, at the same time, using maximum-likelihood methods. All analysis was performed using R version 3.6.0.

To be concrete, here is how the model was implemented to get the statistical results on strategy selection in Fig. 2: let $p_{t,i,k}$ denote the probability of player i selecting cooperation at round t in a session k; let $I_{k \in A}$ be the vector of dummy variables that indicates whether the session k belongs to an experimental treatment A; let ε_k be the

random effects of the session k; let $\varepsilon_{i|k}$ be the random effects of player *i* nested within the session *k*; and let $\varepsilon_{t,i,k}$ be the error. Thus, we have

$$p_{t,i,k} = 1/1\{1 + exp(-z_{t,i,k})\} \quad (1)$$

$$z_{t,i,k} = \beta_0 + \beta_1 I_{k\in A} t + \varepsilon_k + \varepsilon_{i|k} + \varepsilon_{t,i,k} \quad (2)$$

We used a logistic function as the link function for the statistical modeling. The random effects ε_k and $\varepsilon_{i|k}$ are approximated by the normal distribution with mean value zero. Fig. 2 shows the estimated β_1 for each treatment.

To compare the slope of disengaged intervention with that of each other treatment, we modified the equation (2), so that

$$z_{t,i,k} = \beta_0 + (\beta_1 + \beta_2 I^*_{k\in A})t + \varepsilon_k + \varepsilon_{i|k} + \varepsilon_{t,i,k} \quad (3)$$

where $I^*_{k\in A}$ is the vector of dummy variables that indicates whether the session *k* belongs to an experimental treatment *A* instead of the disengaged tie-management. In other words, the statistical model (3) uses the disengaged tie-management as the reference category. Table S1 shows the estimated β_0, β_1, and β_2 and their standard errors.

We may assume that each subject makes their cooperation decision based on their neighborhood environment. We modeled the local influence on cooperation decision-making based on GLMM, so that

$$p_{t,i,k} = 1/1\{1 + exp(-z_{t,i,k})\} \quad (4)$$

$$z_{t,i,k} = \beta_0 + \beta_X X_{t,i,k} + \beta_t t + \varepsilon_k + \varepsilon_{i|k} + \varepsilon_{t,i,k} \quad (5)$$

In model (5), the covariate $X_{t,i,k}$ is the vector of the number of the neighbors of subject *i* at round *t* in the session *k* $x_{t,i,k}$, the rate of cooperators in the neighbors of subject *i* at

round t in the session k $r^C_{t,i,k}$, and the number of cooperators in the neighbors of subject i at round t in the session k $x^C_{t,i,k}$.

In the estimation, we found that the random effect for sessions, ε_k, was nearly zero in our experiment data. Considering the issue of calculation convergence, we removed the session-level random effect from model (5):

$$z_{t,i,k} = \beta_0 + \beta_X X_{t,i,k} + \beta_t t + \varepsilon_i + \varepsilon_{t,i,k} \quad (6)$$

The model still has the random effects for individuals, ε_i. We also confirmed the robustness of the environment effects with the model controlling the status-quo bias (i.e., lagged cooperation), so that

$$z_{t,i,k} = \beta_0 + \beta_X X_{t,i,k} + \beta_t t + \beta_a a_{t-1,i,k} + \varepsilon_i + \varepsilon_{t,i,k} \quad (7)$$

where the covariate $a_{t-1,i,k}$ is a binary variable of whether the subject i chose cooperation at round t-1. Table S2 shows all the estimated coefficients in models (6) and (7). We calculated the cooperation probabilities in Figure 3A by placing the estimated coefficients in model (6) and then model (4).

3. Supplementary references

Peysakhovich, A., Nowak, M.A., and Rand, D.G. (2014). Humans display a “cooperative phenotype” that is domain general and temporally stable. Nature Communications *5*. doi: 10.1038/ncomms5939.

Rand, D.G. (2012). The promise of Mechanical Turk How online labor markets can help theorists run behavioral experiments. Journal of Theoretical Biology *299*, 172–179.

Thomas, K.A., and Clifford, S. (2017). Validity and Mechanical Turk: An assessment of exclusion methods and interactive experiments. Computers in Human Behavior *77*, 184–197.
